# Structure-preserving multivariate hypothesis testing for mass spectrometry imaging and single-cell data

**DOI:** 10.1093/bioinformatics/btag137

**Published:** 2026-03-21

**Authors:** Keziah E Liebenberg, Erin Craig, Robert Tibshirani, Livia S Eberlin

**Affiliations:** Department of Surgery, Baylor College of Medicine, Houston, TX 77030, United States; Department of Biostatistics, University of Michigan, Ann Arbor, MI 48109, United States; Department of Biomedical Data Science and Department of Statistics, Stanford University, Stanford, CA 94305, United States; Department of Biomedical Data Science and Department of Statistics, Stanford University, Stanford, CA 94305, United States; Department of Surgery, Baylor College of Medicine, Houston, TX 77030, United States

## Abstract

**Motivation:**

Mass spectrometry imaging (MSI) and single-cell RNA sequencing (scRNA-seq) offer high-resolution views into tissue and cellular heterogeneity. However, conventional statistical analyses often treat sub-measurements (pixels or cells) as independent, ignoring their nested origin from individual samples. This assumption inflates the effective sample size, increases false discoveries, and undermines biological interpretation. Pixel- or cell-level averaging avoids this but sacrifices spatial or cellular resolution.

**Results:**

We evaluated block-SAM on both simulated and real-world datasets. In DESI-MSI data from kidney, lung, and ovarian tumors, block-SAM consistently identified fewer—but more reliable—differential features compared to traditional-SAM. For example, in the kidney dataset, traditional-SAM identified 569 features between tumor and normal tissue, while block-SAM identified 186—all overlapping but excluding 383 likely false positives. Applied to a metastatic RCC scRNA-seq dataset comparing immune checkpoint blockade (ICB)-treated versus untreated patients, traditional-SAM identified over 19,000 differentially expressed genes in malignant cells; block-SAM reduced this to 19. These results demonstrate block-SAM’s ability to reduce false discoveries while retaining biologically meaningful signals across diverse high-dimensional datasets.

**Availability and implementation:**

All code and data associated with this study are deposited on Zenodo (https://doi.org/10.5281/zenodo.18273497). The *samr* package is freely available on the Comprehensive R Archive Network (CRAN).

## 1 Introduction

Mass spectrometry imaging (MSI) and single-cell RNA sequencing (scRNA-seq) are powerful technologies that have transformed our ability to probe the molecular and spatial landscape of biological tissues at unprecedented resolution. These techniques have uncovered key aspects of tissue heterogeneity (Fasterius *et al.* 2019, Duncan *et al.* 2024), disease progression (Rindler *et al.* 2021, Verbeeck *et al.* 2020), and molecular mechanisms (Chen *et al.* 2019, Yang *et al.* 2024). To extract meaningful biological and medical insights, MSI and scRNA-seq must offer more than visually striking ion images or rich gene expression profiles—they require robust statistical methodologies that can transform raw data into reliable, interpretable findings (Sharman *et al.* 2023).

MSI and scRNA-seq generate large, high-dimensional, and multivariate datasets that pose substantial statistical challenges. In MSI, the integration of rich molecular data with spatial information amplifies this complexity, particularly in untargeted experiments where thousands of molecular features may be compared across sample groups (Tian *et al.* 2018). While the rich molecular and spatial data offer exciting opportunities for new biological discoveries, the curse of high dimensionality substantially increases the risk of false positives (Bemis *et al.* 2019). A second, more fundamental challenge arises from the hierarchical structure of the data: sub-measurements—such as pixels in MSI or cells in scRNA-seq—originating from shared biological sources or a single patient sample are not statistically independent (Jones *et al.* 2011). Whether it is appropriate to treat these units as independent measurements depends on the scientific question. For within-sample comparisons (e.g., necrotic versus viable tumor tissue regions, or cancer cells versus immune cells), pixel- or cell-level analysis may be valid. However, when comparing between the patient groups (e.g., cancer versus normal tissues), failing to account for the shared origin of sub-measurements can bias statistical inference by inflating the effective sample size and underestimating the variance.

A common approach to address this challenge in MSI is pixel-wise averaging, by which the relative intensity of a molecule is averaged across all pixels within a sample or a region-of-interest before statistical testing. However, this method defies the purpose of imaging as it sacrifices the spatial heterogeneity captured by MSI, reducing the molecular insights gained and making the analysis more akin to traditional shotgun mass spectrometry (Hu and Laskin 2022). Despite the technological sophistication and the advances in data acquisition methods in MSI and scRNA-seq, progress in the statistical pipelines for data analysis has not kept pace, with no accepted standardized approach for hypothesis testing that both preserves sub-unit resolution and respects sample structure (Sharman *et al.* 2023). To address these limitations, we present a novel hypothesis testing framework, block-SAM (Significance Analysis of Microarrays), designed specifically for structured, high-dimensional biological data. Block-SAM retains the molecular and spatial richness of MSI and the cellular resolution of scRNA-seq, while ensuring statistical rigor by accounting for the nested structure of pixel- and cell-level measurements. The proposed method is a modification of the popular SAM method for analysis of unblocked high-dimensional data.

## 2 Materials and methods

### 2.1 Statistical rationale

From a mathematical and statistical perspective, why is it incorrect to treat pixels from MSI experiments or cells from scRNA-seq as independent observations? After all, these technologies deliver unprecedented resolution at the cellular and molecular level, and investigators should be able to leverage the richness of these data to strengthen statistical inferences. Simply put, statistical significance depends on the observed difference between groups relative to the variability expected by chance. For example, in a simple two-sample comparison, the *t*-test statistic is often computed as follows:


$$t=\;\frac{{\bar{X}}_{1}-\;{\bar{X}}_{2}}{\sqrt{\frac{s_{1}^{2}}{{n_{1}}^{2}}+\frac{s_{2}^{2}}{{n_{2}}^{2}}}}$$

where ${\bar{X}}_{i}$, $s_{i}$, and $n_{i}$ denote the mean, sample variance, and sample size for group i, respectively. Treating pixels as independent (i) inflates the sample size ${(n}_{1}\;or\;n_{2})$, as there are a large number of pixels or cells originating from often a much lower number of independent samples and (ii) underestimates the variance ($s_{1}^{2}$ or $s_{2}^{2}$) by failing to account for similarity among pixels within each sample. Together, these issues make t artificially large, exaggerating the apparent power to detect differences between groups. As a result, even tiny differences can become statistically significant. This issue further extends to nonparametric methods. The Wilcoxon rank-sum test (or Mann–Whitney *U*-test), a widely used nonparametric alternative, also relies on sample size to estimate variability. The test statistic is based on the ranks of the combined data, and under the null hypothesis, the variance is similarly calculated using group sample sizes. Therefore, both parametric and nonparametric methods are vulnerable to increased false positive rates when applied directly to datasets with a nested structure.

**Figure 1:**
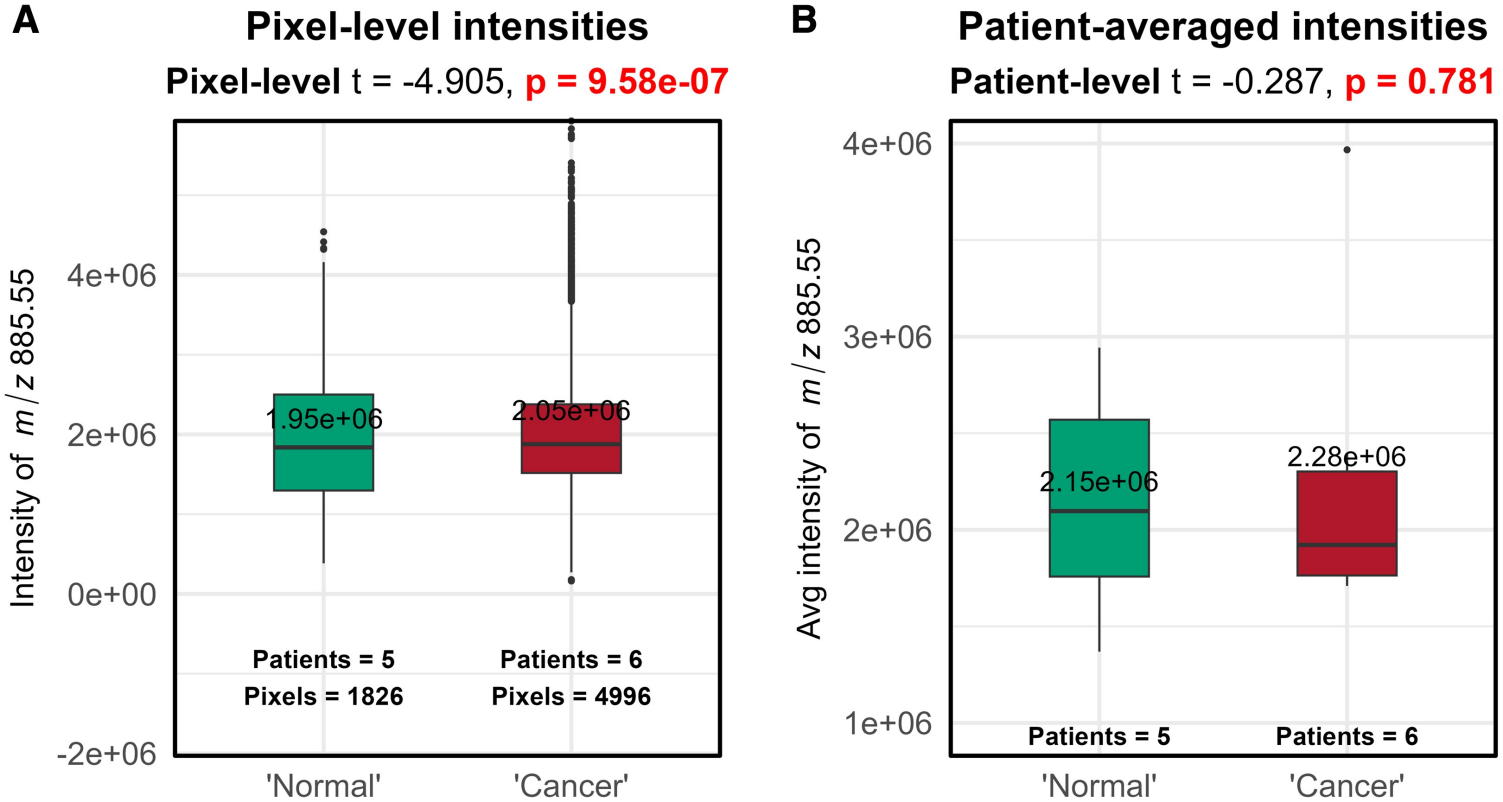
Statistical rationale: comparing pixel- versus patient-level statistics. (A) Box plot of the distribution of intensity values for *m*/*z* 885.549 per pixel across two “simulated” groups (“Normal”: mean = 1.95 × 10^6^; versus “Cancer”: mean = 2.05 × 10^6^). (“Normal”: *n* = 5 patients, 1826 pixels; “Cancer”: *n* = 6 patients, 4996 pixels). A pixel-level *t*-test treating every pixel as an independent observation reveals a significant difference between groups (*P *= 9.58e−07). (B) Box plot of the distribution of intensity values for *m/z* 885.549 per patient across the two “simulated groups” (“Normal”: mean = 2.15 × 10^6^; versus “Cancer”: mean = 2.28 × 10^6^). A patient-level *t*-test shows no significant difference (*P *= 0.781).

To illustrate this, a real MSI dataset consisting of 11 samples solely from cancerous tissues was used to create two artificial groups with no true differences between them. Five of these samples were randomly labeled as a “normal” group (total of 1826 pixels), despite all originating from diseased tissue, and the remaining six labeled as “cancer” group (total of 4996 pixels) (Fig. 1A). Since the group labels were artificially assigned, no true systematic differences exist between the “normal” and “cancer” groups due to disease state. To highlight a biologically relevant example, we selected a representative membrane phospholipid (*m/z* 885.549) commonly detected in MSI data from human tissues. We compared the intensity of *m/z* 885.549 between simulated groups using two statistical approaches. The first was a pixel-level *t*-test, which treated each pixel as an independent observation. The second was a patient-level *t*-test, conducted on the average intensity per patient, thereby preserving the correct unit of biological replication. As shown in Fig. 1, the pixel-level *t*-test yielded a statistically significant difference between the groups (*P *= 9.58e−07), while the patient-level test found no significant difference (*P *= 0.781). This discrepancy arises from the artificially inflated sample size in the pixel-level test, which ignores the non-independence of pixels from the same image. By contrast, the patient-averaged approach correctly accounts for the hierarchical structure of the data and avoids spurious statistical significance.

Although single-cell and imaging techniques yield vastly more measurements per sample than traditional bulk approaches, these additional units do not correspond to additional biological replication. Instead, they often give the illusion of increased statistical power while inflating false positive rates by boosting the apparent sample size and decreasing sample variance. In this way, the higher resolution of these technologies can undermine statistical reliability when the nested data structure is ignored. Despite widespread use however, many existing tools do not properly correct for this issue.

### 2.2 Related work

Statistical methods are widely used in the field of MSI to identify patterns, reduce dimensionality, and detect differences across groups within high-dimensional molecular datasets. Conventional multivariate techniques such as principal component analysis (PCA), clustering algorithms (e.g. hierarchical and *k*-means) (Verbeeck *et al.* 2020), partial least squares discriminant analysis (PLS-DA), factorization methods, and various supervised statistical and machine learning approaches are typically used to perform dimensionality reduction and separate sample groups (Tian *et al.* 2018, Sharman *et al.* 2023). Following which, univariate or multivariate statistical tests are frequently employed to compare the abundances of individual molecular features across different sample groups. Nonparametric methods include the Wilcoxon and Kruskal–Wallis tests (Jones *et al.* 2011), while parametric approaches such as the *t*-test and one-way ANOVA are also commonly applied (Hu and Laskin 2022). These tests, however, are often performed at the pixel level under the assumption of independence among measurements.

A variety of open-source and commercial software platforms have been developed to facilitate visualization, data pre-processing, and statistical analysis of MSI datasets. Several of these platforms, including SCiLS, MSiReader, OpenMSI, OmniSpect, MassImager, LipostarMSI, and MassExplorer, offer built-in statistical analysis capabilities (Bond *et al.* 2017). Custom R and Python packages have also been designed for multivariate statistical analysis of MSI data, including Cardinal, massPix, and HIT-MAP (Tian *et al.* 2018). While these tools offer robust capabilities for visualization, image segmentation, and preprocessing, their statistical modules often rely on conventional approaches that do not account for the correlation between spatially adjacent pixels and the hierarchical organization of pixels within tissue samples. Altogether, the statistical implications of pixel-level dependence remain largely underexamined in the MSI literature, despite their potential to substantially impact biological findings.

In the field of scRNA-seq, differential expression (DE) analysis is a central statistical approach, allowing for the identification of genes that differ between cell clusters or experimental conditions. Commonly used statistical methods include the Wilcoxon rank-sum test, *t*-test, MAST, Monocle, SCDE, Seurat, Scanpy, likelihood ratio test and linear mixed models (LMMs) and limma (Dal Molin *et al.* 2017, Squair *et al.* 2021). Furthermore, adaptations of bulk RNA-seq tools such as DESeq2 (Love *et al.* 2014) and edgeR (Robinson *et al.* 2010) have also been employed to scRNA-seq data in a pseudobulk manner, whereby raw counts are summed across cells per sample (Finak *et al.* 2015, Guo *et al.* 2024). While these approaches prevent the treatment of cells as independent measurements, they sacrifice the single-cell resolution and as such, the output resembles that of a bulk RNA-seq experiment. In contrast, methods such as Monocle, MAST, and SCDE have been specifically developed for scRNA-seq data and adapted to combat challenges such as drop out events and sparsity of the datasets (Finak *et al.* 2015, Dal Molin *et al.* 2017, Squair *et al.* 2021).

Despite widespread adoption, several limitations have been identified in DE methods. Firstly, studies have reported that different statistical methods often produce inconsistent results, identifying varying numbers of differentially expressed genes even when applied to the same datasets (Dal Molin *et al.* 2017). More concerningly, large false positive rates have been observed across multiple studies (Dal Molin *et al.* 2017, Soneson and Robinson 2018, Wang *et al.* 2019, Squair *et al.* 2021, Nguyen *et al.* 2023), in which genes have been identified as differentially expressed, even when no biological differences between groups exist. A known bias toward highly expressed genes has also been highlighted, where minor shifts in expression are incorrectly identified as differential, even though they are not biologically meaningful (Soneson and Robinson 2018, Squair *et al.* 2021). These issues support the statistical rationale previously exemplified in Fig. 1. The inflated false positive rates reported for single-cell DE methods have been primarily attributed to their failure to account for biological replication, leading to an underestimation of gene expression variance. While this conclusion is valid, another critical gap should be noted: current single-cell DE methods not only ignore between-sample replication but also fail to model the ‘within-sample correlation’ that arises from measuring many cells derived from the same sample or patient. This additional layer of pseudoreplication further contributes to inflated significance levels.

Although the data and statistical methods discussed above are well-established and widely used in biomedical research, they share a critical limitation when applied to high-dimensional, multivariate datasets with a nested structure—such as MSI and scRNA-seq. Both data types exhibit high sparsity, noise, non-normal distributions, and substantial biological heterogeneity, and involve repeated, non-independent sub-unit measurements (pixels or cells) derived from a limited number of samples (Nguyen *et al.* 2023). It is therefore clear that new methods and statistical analysis pipelines need to be developed and refined within the fields of MSI and scRNA-seq.

Here, we introduce block-SAM, a statistical framework that extends SAMs to account for hierarchical data structure. Block-SAM performs permutation-based hypothesis testing while preserving biologically accurate groupings, thereby enabling statistically valid feature selection without discarding pixel- or cell-level resolution. By respecting the nested data structure, block-SAM provides more reliable control of false discovery rates (FDRs) in high-resolution spatial and single-cell data. The method builds upon the existing infrastructure of the samr R package, allowing for practical implementation within familiar analysis workflows (Tusher *et al.* 2001, Tibshirani *et al.* 2018).

### 2.3 Block permutation tests for biologically nested data

#### 2.3.1 Implementation for targeted analyses

Suppose we have MSI data consisting of many pixels per patient, and we aim to conduct a targeted analysis to determine whether the relative abundance of a specific molecule differs between two groups: “normal” and “cancer.” A straightforward approach might be a *t*-test comparing pixels from each group, treating them as independent and assigned to one of two categories (normal or cancer) without accounting for within-patient relationships. This assumption is incorrect but let us examine the consequences of making it.

**Figure 2:**
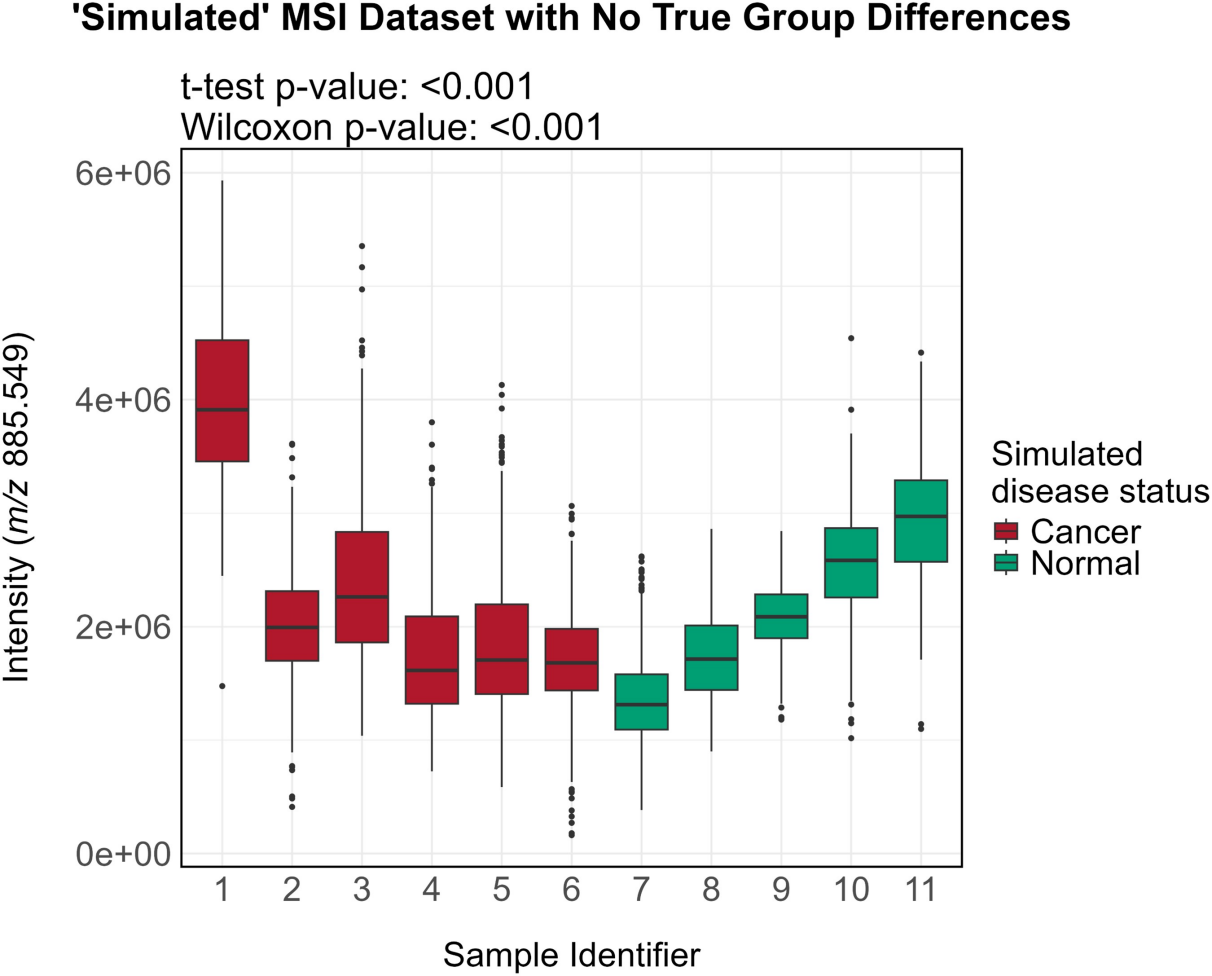
“Simulated” MSI data for targeted analysis. Boxplot of the relative abundances of *m/z* 885.549 across “simulated” groups. All samples in this dataset originate from MSI data of cancer tissue. For illustrative purposes, half of the images were randomly relabeled as “normal”, despite all being cancer tissues. Since the group assignments were arbitrary, no true systematic differences exist between the “normal” and “cancer” groups. However, both *t*-test and Wilcoxon test return *P*-values were below .001.

To illustrate this, we used the same MSI dataset described in the statistical rationale section, comprising 11 cancer tissue samples, randomly divided into two artificial groups with no true biological differences. As before, to highlight a biologically relevant feature, we selected a representative membrane phospholipid (*m*/*z* 885.549). The distribution of *m*/*z* 885.549 intensities for all pixels is visualized in Fig. 2, with pixels grouped by their respective patient samples. Although all data within the assigned groups are from cancer tissues, expected variations between individuals remain. These differences arise from factors such as biological variation, instrument settings, environmental conditions, practitioner-specific techniques, and others.

In this example dataset, when incorrectly considering the pixels as independent measurements, the difference in normalized intensities of *m/z* 885.549 between “normal” and “cancer” tissue was 4.10 × 10^5^. Since the group labels were artificially assigned, this value does not reflect a true systematic difference between the two groups. Yet, both a *t*-test and a Wilcoxon test using *n* = 6822 (total number of pixels) return *P*-values below .001, highlighting the risk of incorrectly assuming pixel independence. This statistical oversight can lead to the detection of apparent differences between groups when none exist, driven solely by inter-individual variation.

To address the non-independence of pixels originating from the same patient, a hypothesis test that respects this structure is necessary. A commonly used method is the block permutation test (Pitman 1937, Pesarin 2001, Good 2005), illustrated by the following example. The initial hypothesis tests asked: if there were no true difference in intensities between pixels from normal and cancer samples, what is the probability of observing an absolute difference in intensities of at least 4.10 × 10^5^? However, the correct formulation should account for within-image correlations: if there were no true difference in intensities between pixels from normal and cancer samples, and pixel-level correlations within images are considered, what is the probability of observing an absolute difference in intensities of at least 4.10 × 10^5^?

Suppose many datasets were generated under the null hypothesis, where (i) there is no true difference in pixel intensities between normal and cancer samples and (ii) pixels within images are correlated. The goal would be to assess how similar the observed dataset is to these null datasets. This can be done by calculating how often the absolute difference in intensities within the null datasets exceeds 4.10 × 10^5^. For example, if only 1% of the null datasets showed an absolute difference greater than 4.10 × 10^5^, it would suggest that the observed dataset is unlikely to have arisen under the null hypothesis (corresponding to a *P*-value of .01). Conversely, if 90% of the null datasets exceeded this threshold, it would indicate that the observed dataset is consistent with the null hypothesis (*P*-value of .90).

This framework forms the basis of a permutation test. To perform such a test, multiple datasets satisfying the null hypothesis are needed. Permutation tests generate these datasets directly from the original data by randomly permuting the group labels (“normal” or “cancer”) across images. This process has two critical effects: (i) It eliminates any systematic difference between healthy and sick groups by disrupting the original group assignments. (ii) It preserves the within-image correlation structure, since all pixels from the same image remain grouped together, thus avoiding the incorrect assumption that pixels are independent.

**Figure 3:**
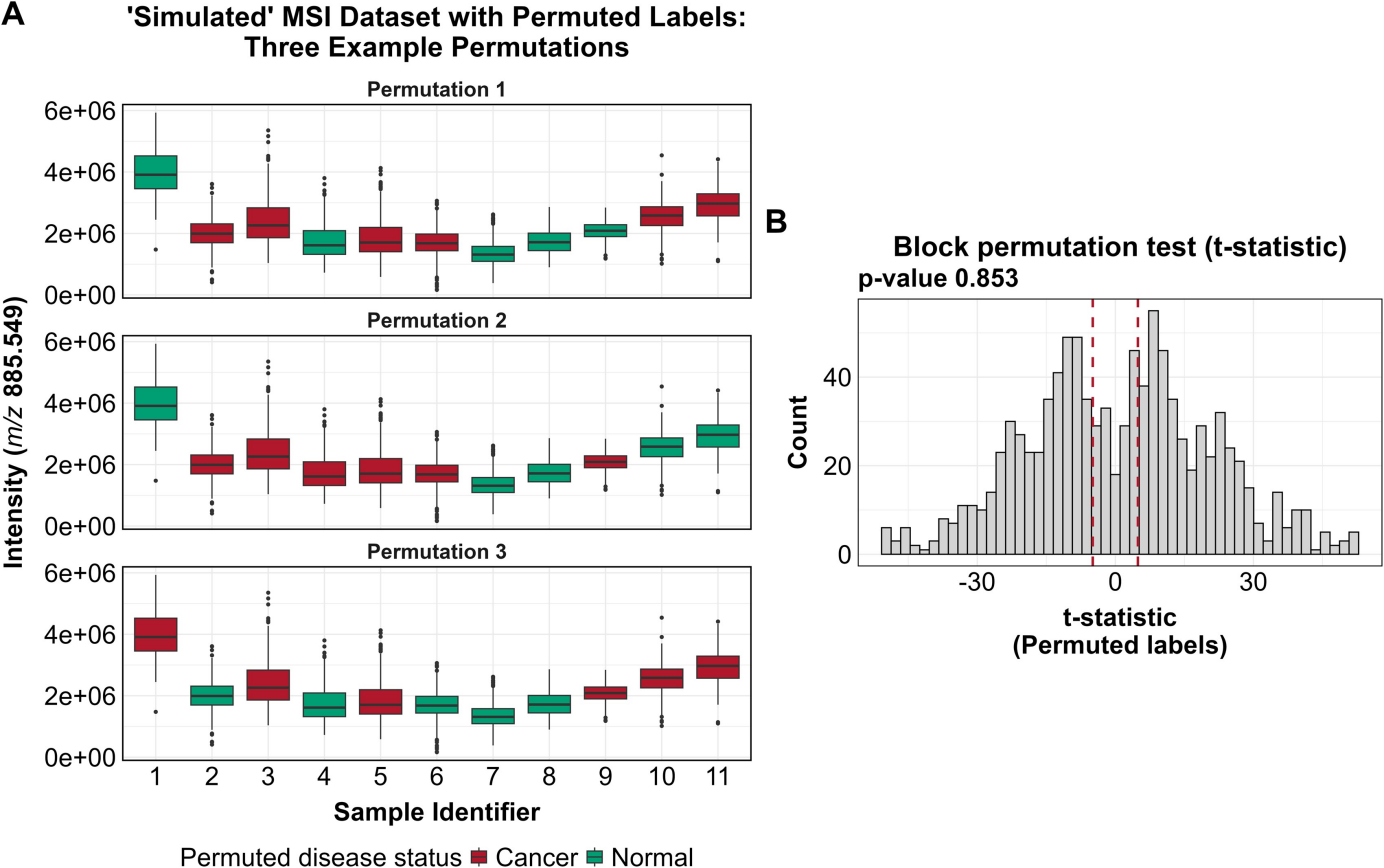
Block permutation test. (A) Example block permutations of “simulated” MSI dataset. In each permutation, pixels are randomly relabeled as “cancer” or “normal” with the constraint that all pixels from the same sample are relabeled together. For instance, in the first permutation, all pixels from Sample 1 are assigned the “normal” label, whereas in the second and third permutations, all pixels from Sample 1 are assigned the “cancer” label. (B) Permutation test results. The empirical distribution shows the differences in relative abundances obtained across all permutations. The two vertical lines indicate the observed difference from the original dataset and its negative. The *P*-value is calculated as the proportion of permutations in which the absolute value of the shuffled difference exceeds the absolute value of the observed difference.

To perform the permutation test, image labels are randomly permuted a large number of times (e.g., 1000 permutations), and a *t*-test statistic is recalculated for each permutation. Figure 3A illustrates three examples of such permutations. The *P*-value is the fraction of permutations in which the absolute value of the shuffled *t*-statistic exceeds the observed difference. In this example, the block permutation *P*-value is 0.853, correctly leading to a failure to reject the null hypothesis that no difference exists between groups. This result is illustrated in Fig. 3B.

Note that a two-sided hypothesis test was performed, as there was no prior expectation regarding whether normal or cancer samples would exhibit a higher relative abundance. Accordingly, the permutation test evaluates whether the shuffled differences are either greater than the observed difference or smaller than its negative. This is illustrated in Fig. 3B, where the observed difference and its negative are marked by two vertical lines.

#### 2.3.2 Implementation for untargeted analyses

Now consider MSI data in the context of untargeted analysis, where the goal is to test for differences across hundreds or thousands of molecular features. A common approach is to perform a statistical test (e.g., a *t*-test) for each molecule and then either (i) adjust the resulting *P*-values for multiple comparisons or (ii) apply FDR control to identify features with statistically significant differences at a specified threshold (e.g., 1% FDR). However, each of these individual tests is subject to the same problem identified in the targeted analysis: pixel-level measurements within the same image are not independent. Fortunately, the solution is the same. For each molecule, a permutation test is performed that preserves the grouping of pixels by image, thereby accounting for within-image correlation.

To evaluate data from untargeted analysis, the SAMs method (Tusher *et al.* 2001) can be used, along with its corresponding R package, samr (Tibshirani *et al.* 2018). SAM evaluates each molecule for differences in relative abundance between groups (e.g., normal versus cancer) using a moderated test statistic and permutation-based estimation of the null distribution and calculation of the FDR. However, standard SAM permutes labels across all individual observations, assuming independence—a limitation in pixel-based or single-cell data. To address this, we developed block-SAM, an extension of the original SAM framework that now preserves biological grouping (e.g., by patient or tissue section) during permutation. This modification enables statistically valid feature selection in structured, high-resolution datasets while retaining the interpretability and FDR control of the original method.

Systematic benchmarking was conducted to characterize block-SAM’s computational requirements across varying dataset scales. Using subsampled versions of a kidney DESI-MSI dataset, (normal samples, *N* = 16; renal cell carcinoma (RCC) samples, *N* = 39) computational runtime and memory usage of block-SAM was evaluated by varying the number of patients per group (5–39), features (50–500), and permutations (100–1000). Runtime and memory increased smoothly with problem size, with the smallest setting (five patients per group, 50 features, 100 permutations) taking ∼0.9 s and using ∼7.9 MB, and the largest setting (39 patients per group, 500 features, 1000 permutations) taking ∼6.67 min and using ∼594 MB. Block-SAM completed in minutes—e.g., 20 patients per group with 200 features required ∼2.19 min (500 permutations) or ∼3.10 min (1000 permutations) and ∼213–386 MB peak memory—suggesting that the main driver of compute cost is the number of permutations.

To illustrate the utility of block-SAM, we simulate data under the null hypothesis of no true difference between the “normal” and “cancer” groups. This simulation is based on the same dataset used in the untargeted analysis, with one key change: instead of focusing on a single molecule, all 1295 molecular features detected in the MSI data are included. As before, the group labels are randomly assigned, ensuring that no true differences exist between the two groups. The SAM function was then applied to the simulated data. Importantly, the argument intactBlocks = TRUE is specified to ensure that permutations respect the grouping of pixels within images—preserving the correlation structure inherent to MSI data. The input to SAM includes a matrix x, where each row represents a molecule and each column a pixel, and a vector group.names, which indicates the group assignment (“normal” or “cancer”) for each pixel, based on the patient of origin.

**Figure 4:**
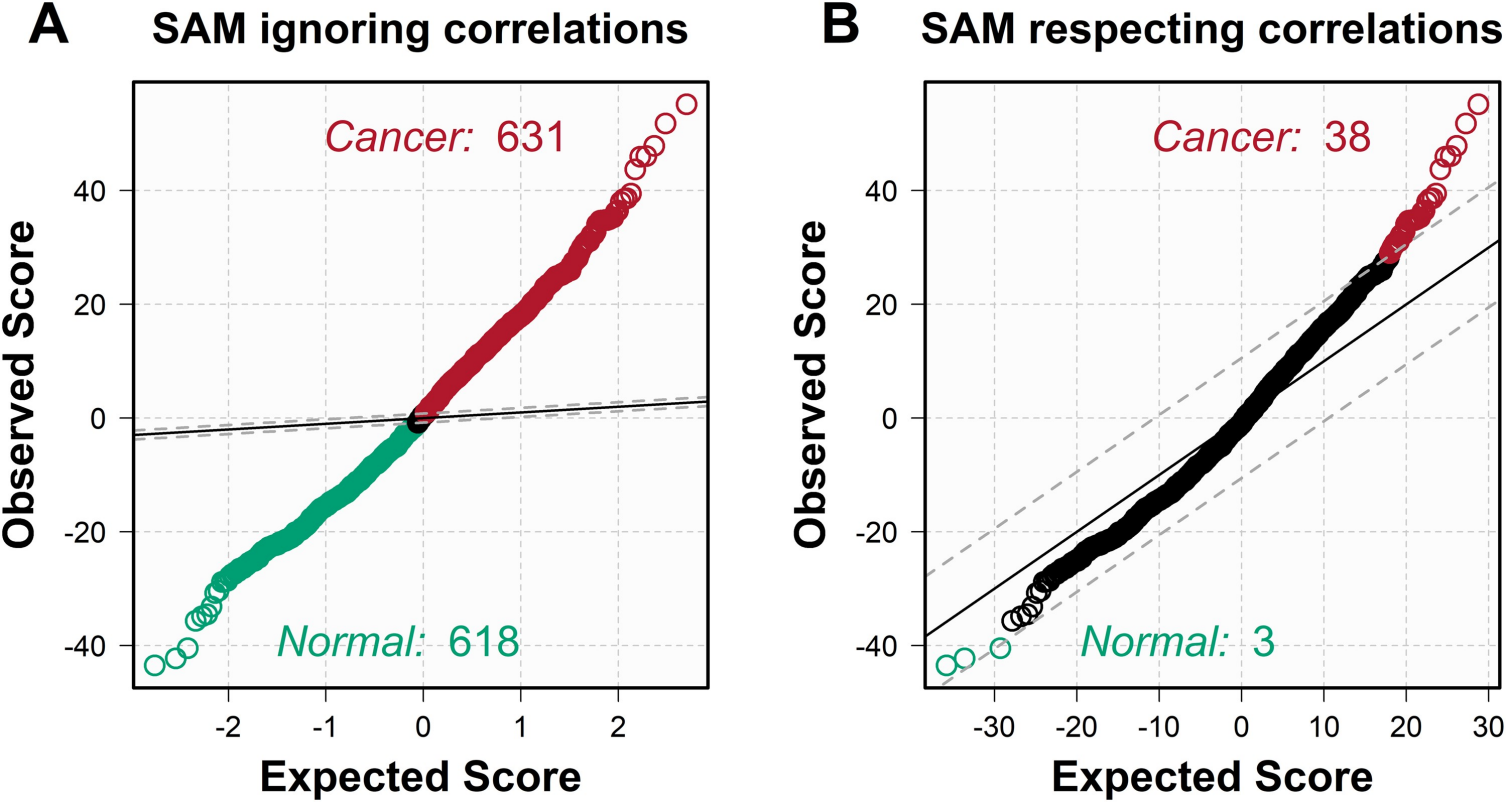
Comparison of SAM results. (A) SAM results without block permutation. When pixel-level dependencies are ignored, SAM erroneously identifies 1249 molecules as significantly different between groups—illustrating the severe inflation of false positives that results from treating pixels as independent observations. (B) SAM results with block permutations. When block permutation tests are used, SAM identifies only 41 molecules with statistically significant differences between the healthy and sick groups, reflecting appropriate control of the false discovery rate under the null hypothesis.

Running SAM yields a list of molecules with significantly higher relative abundance in either the “normal” or “cancer” group. Figure 4A displays the number of molecules selected in the “normal” group (green) and the “cancer” group (red), based on a comparison between the observed test statistics and their expected values under the null hypothesis. A substantial divergence between observed and expected values indicates evidence against the null hypothesis. In this example, where no true group differences exist, when original SAM is run ignoring the correlation among pixels within images (intactBlocks = FALSE), it erroneously identifies 1249 significant molecules of the total 1295 molecules as significant (92.6%) (Fig. 4A). In contrast, block-SAM identifies only 41 significant molecules out of 1295 tested (3.2%)—an acceptable number of false positives given the specified 1% FDR threshold (Fig. 4B). This result echoes the findings from the targeted analysis: failing to account for within-image correlation leads to a dramatically inflated false positive rate.

### 2.4 Simulation-based validation of block-SAM

#### 2.4.1 Evaluation of type I error control and power

**Figure 5:**
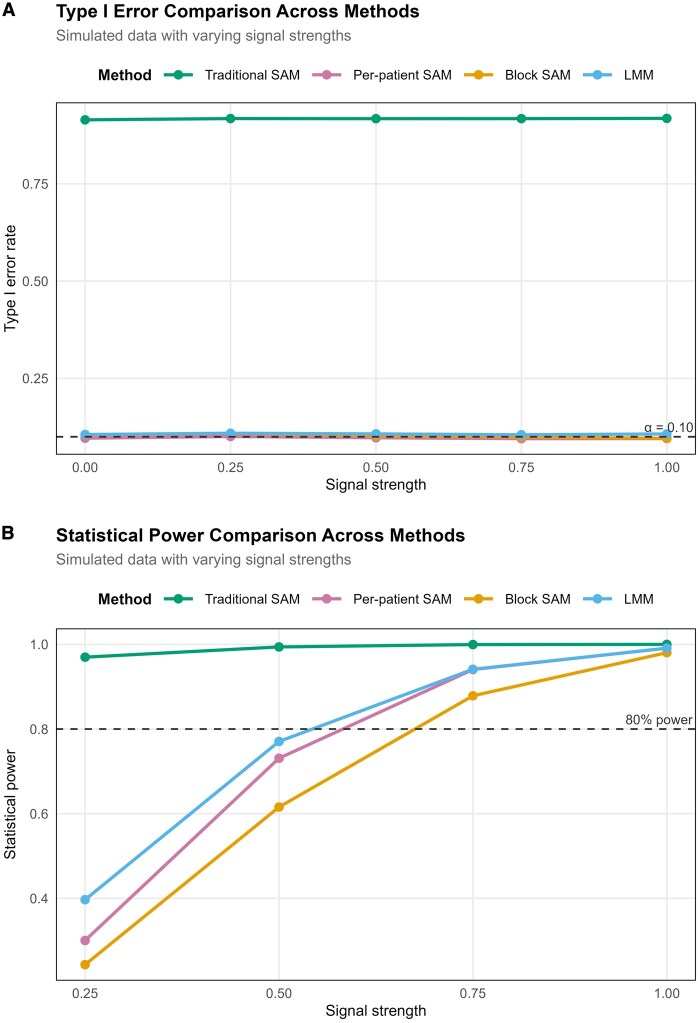
Method comparison on a null-difference kidney DESI-MSI simulation. (A) Type I error control. Type I error is well controlled for all methods except traditional-SAM, which incorrectly treats pixels from the same image as independent and therefore fails to account for within-image correlation. (B) Statistical power across methods versus signal strength. Power increases with signal strength for per-patient SAM, block-SAM, and LMM, with LMM achieving the highest power across the range of effects.

**Figure 6:**
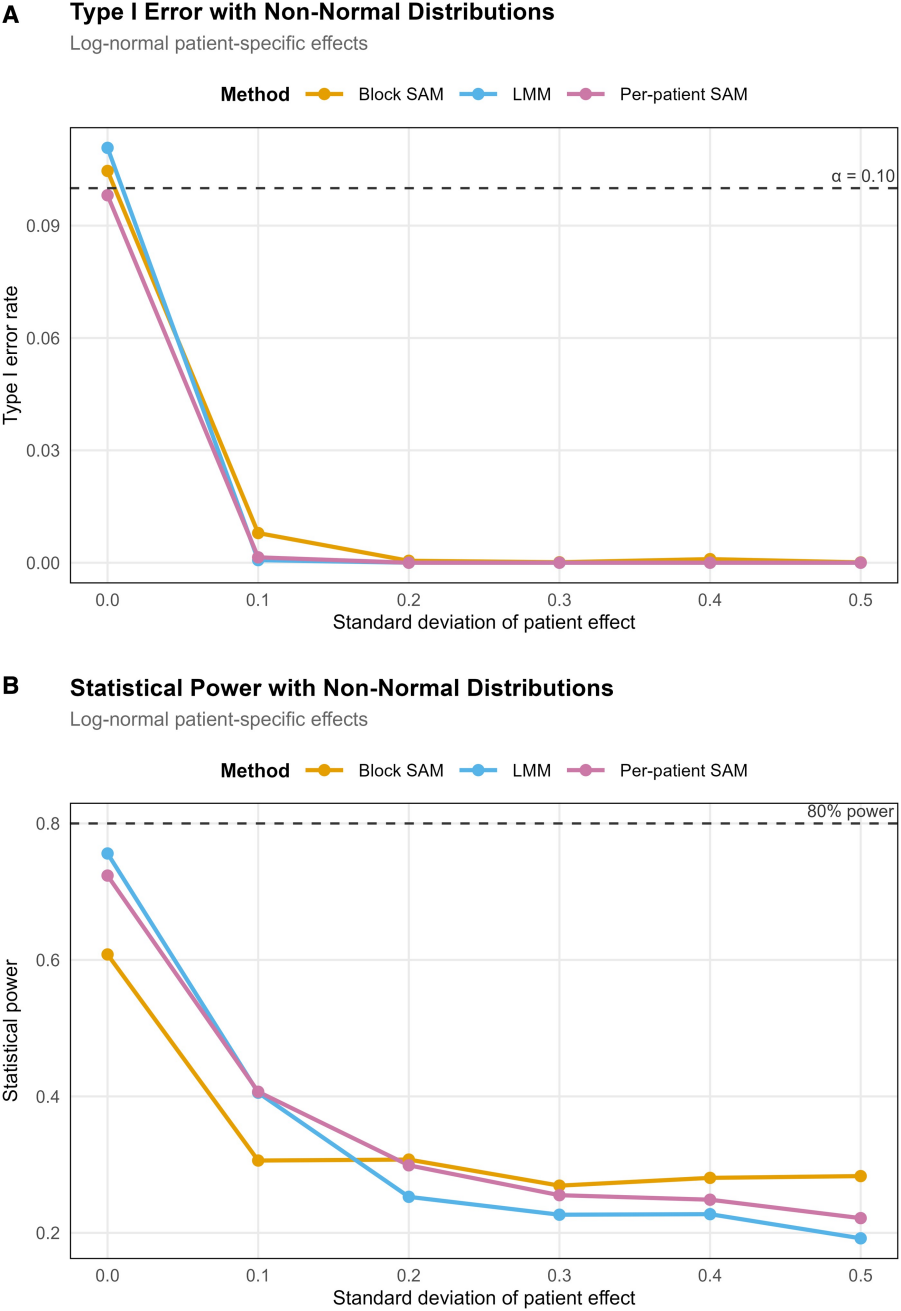
Robustness to non-normal patient-specific effects. (A) Type I error rate. Type I error remains well controlled for block-SAM, per-patient SAM, and LMM as the magnitude of the (log-normal) patient-specific effect increases (*x*-axis: standard deviation of the patient effect). The dashed horizontal line indicates the nominal level (*α* = 0.10). (B) Statistical power. Block-SAM retained the greatest power across an increase in patient-specific effects compared to the alternative approaches.

To create a ground truth dataset for benchmarking, a “null-difference” dataset was generated utilizing a kidney DESI-MSI dataset. The “null-difference” dataset was created where the patients from the RCC class (39 samples; 36 431 pixels; 613 *m/z* features) of the kidney DESI-MSI dataset were randomly split into two pseudo-groups labeled “RCC” (*N* = 20) and “Normal” (*N* = 19). This approach preserves within-patient structure while eliminating true between-patient biological signals. To assess power, signal was introduced into 50 randomly selected features by shifting values in the RCC pseudo-group by a multiple of the feature’s standard deviation (labeled “Signal strength” in Figs 5 and 6). Type I error was defined as the proportion of null features incorrectly rejected at *α* = 0.1, and power as the proportion of signal features correctly detected. This simulation framework was applied across traditional-SAM, per-patient SAM, block-SAM, and LMMs to directly compare method performance under identical conditions. A total of 100 repeated simulations were conducted for each signal strength, and the mean values are reported in the line graphs in Figs 5 and 6.

Traditional-SAM was found to be the only approach without the desired type I error; this is expected as it ignores patient effects. Among the three valid models, LMM was the most powerful, followed by per-patient SAM and then block-SAM. Importantly, LMM assumes the patient-specific effects are normally distributed, and this extra assumption improves power when it is correct. Block-SAM, however, provides a robust nonparametric alternative that maintains valid inference without distributional assumptions, making it well-suited for the complex correlation structures common in MSI data.

To address within-patient dependence, we compared block-SAM with per-patient SAM, which aggregates pixels to patient-level means, and found that when pixel counts are balanced and patient-level variance is used, the two approaches are effectively equivalent (Fig. S1, available as supplementary data at *Bioinformatics* online).

For further benchmarking of block-SAM, algorithmic reproducibility was assessed by rerunning the method 10 times with different random seeds and observed near-identical feature call sets (mean pairwise Jaccard = 0.979; Fig. S2, available as supplementary data at *Bioinformatics* online).

#### 2.4.2 Evaluation of robustness to normality assumption

To assess the robustness to violations of the LMM normality assumption, skewed patient-level random effects were injected into the data. The signal strength was fixed at 0.5, and log-normal random effects were added with increasing degrees of skewness (skew parameter *σ* = 0, 0.1, 0.2, 0.3, 0.4, 0.5), then centered within each group to preserve the null hypothesis. When *σ* = 0, no additional effects were added (baseline). Traditional-SAM was not included in this analysis as it is not a valid method for DESI-MSI data. As before, 100 simulations were run for each skew parameter.

All methods were found to be overly conservative (type I error close to 0) and showed a substantial loss of power due to the added patient-specific effects. Notably, the power loss was most pronounced for LMM and per-patient SAM. For LMM, the power loss reflects its assumption that random effects follow a normal distribution, therefore when the true patient effects are log-normal (skewed), this assumption reduces the model’s ability to detect group differences. In contrast, block-SAM demonstrated notably greater robustness as it makes no assumptions regarding the *distribution* of patient effects. Block-SAM’s ability to maintain power in the presence of skewed patient effects make it particularly well-suited for MSI datasets, where the hierarchical structure and non-normal variability are common features of the data.

Block-SAM power was further evaluated across a range of patient sample sizes, demonstrating improved power with larger cohorts and inherent limitations at very small n due to the finite number of unique permutations (Fig. S3, available as supplementary data at *Bioinformatics* online).

## 3 Implementation

### 3.1 Application of block-SAM to published datasets

#### 3.1.1 A MSI example

To showcase the effectiveness of block-SAM to evaluate MSI data, we applied it to three previously published desorption electrospray ionization (DESI) MSI datasets: kidney (Zhang *et al.* 2020), ovarian (Sans *et al.* 2017), and lung (Bensussan *et al.* 2020) tissue datasets.

**Figure 7:**
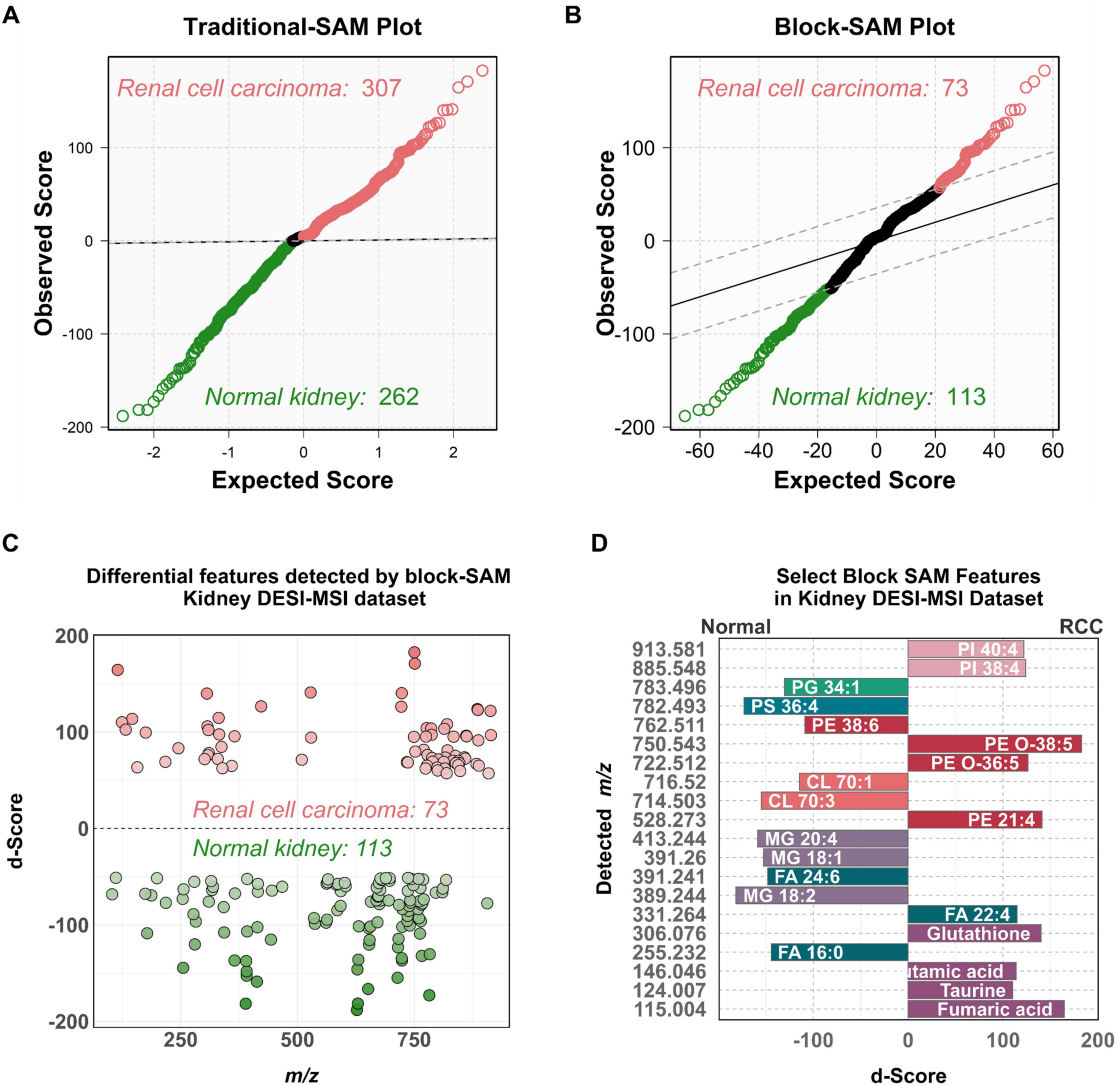
Traditional- and block-SAM analysis of kidney MSI data. (A) Traditional-SAM plot. Features associated with RCC (red, *n *= 307) and normal kidney tissue (green, *n *= 262) deviate from the null distribution, while non-significant features (black) fall within the threshold bounds (dashed lines). (B) Block-SAM plot. Fewer features are identified as significant using block permutation. RCC-associated features (red, *n *= 73) and normal-associated features (green, *n *= 113) deviate from the null. (C) Distribution of differential *m/z* features detected by block-SAM. Features with positive *d*-scores (above dashed line) are elevated in RCC; those with negative *d*-scores are elevated in normal tissue. (D) Tentative annotations of selected block-SAM features. Labels follow the format: molecular class abbreviation, carbon count: double bonds. Abbreviations: CL, cardiolipin; MG, monoacylglycerol; PG, phosphatidylglycerol; PS, phosphatidylserine; FA, fatty acid; PE, phosphatidylethanolamine; PI, phosphatidylinositol; SM, small molecule.

The first dataset was a 2020 DESI-MSI study of 71 human kidney tissue samples, including 16 normal kidney (total of 20 396 pixels) and 39 RCC (total of 36 431 pixels) tissues. Each tissue type was hypothesized to exhibit a distinct metabolic profile, reflected in the relative abundance of specific molecular ions. This dataset presents an ideal test case for block-SAM, as it involves complex, pixel-structured MSI data and multiple biological groups. When applying the traditional-SAM, a total of 569 metabolic features were identified as significantly different between normal kidney and RCC tissues (Fig. 7A). Of these, 307 were associated with RCC, while 262 were associated with normal kidney tissues. However, as previously explained, this approach does not account for the pixel structure of the data, thereby potentially limiting the biological relevance of the identified features. Furthermore, as can be seen in Figure 7A, the significance threshold calculated by traditional-SAM does not display the desired statistical rigidity, leading to excessive false positives. Therefore, when applied to MSI data, traditional-SAM lacks the necessary robustness to provide sufficient protection against spurious findings.

Utilizing block-SAM, a total of 186 metabolic features were selected as statistically significantly different between normal kidney and RCC tissues (Fig. 7B). Of these, 73 features were weighted toward RCC and 113 weighted toward normal kidney tissues. Similar to traditional-SAM, a greater number of features were found to be present in a higher relative abundance in RCC tissues compared to normal kidney. However, block-SAM resulted in a substantial reduction in the total number of features identified as statistically different between the two tissue types. This result clearly demonstrates how utilizing block-SAM effectively lowers the false positive rate of the method.

Interestingly, as seen in Table 1, all 186 features identified by block-SAM were identified within the 569 features selected by traditional-SAM. This observation highlights that although traditional-SAM selected statistically significant features, its elevated FDR also resulted in its selection of 2.1 times more non-significant features. This comparison highlights the enhanced statistical robustness of block-SAM when applied to MSI datasets. Importantly, by limiting the number of selected features, block-SAM reduces the time required for chemical annotation of features. This allows for a more focused analyses of significant molecular changes between the tissue types compared.

**Table 1:** Comparison of the number of statistically significant features identified by traditional-SAM and block-SAM in the kidney DESI-MSI dataset.

Feature category	Number of features
Total features (traditional-SAM)	569
Total features (block-SAM)	186
Common	186
Unique to traditional-SAM	383
Unique to block-SAM	0

For example, the distribution of the *m/z* features selected as significant by block-SAM can be seen in Fig. 7C. Furthermore, the molecular annotations of a subset of differential features between normal kidney and RCC tissues are presented in Fig. 7D. Normal kidney tissues exhibited a higher abundance of select monoacylglycerol species, fatty acids, cardiolipins, and phospholipids. Notably, RCC tissues exhibited a higher abundance of metabolites such as glutathione, glutamic acid, fumaric acid, and taurine. This finding suggests increased activity in various energy metabolism pathways known to be dysregulated in cancer cells, including the TCA cycle and the metabolism of methionine, S-adenosyl methionine, cysteine, and taurine. Understanding these metabolic distinctions is essential, as they can reveal key biological pathways driving disease progression and identify potential therapeutic targets. Block-SAM can therefore be a powerful tool for leveraging the biological insights gained from MSI experiments.

The application of block-SAM to the remaining datasets provided similar results and reinforced the key trends observed in the kidney dataset. In the lung adenocarcinoma dataset, traditional-SAM identified 397 features, and block-SAM identified 162 features. In the lung squamous cell carcinoma dataset, traditional-SAM identified 472 features, and block-SAM identified 250 features. Lastly, in the ovarian cancer traditional-SAM identified 771 features, while block-SAM identified 319 features. Detailed results are shown in Figs S4–S9, available as supplementary data at *Bioinformatics* online. The consistent performance of block-SAM across these tissue types and datasets reinforce the value and robustness of the block-SAM method for MSI data statistical analysis.

#### 3.1.2 A scRNA-seq example

We next evaluated the utility of block-SAM to scRNA-seq data using a published scRNA-seq dataset from Bi *et al.* (2021) available through the Broad Institute’s Single Cell Portal. The study characterized the transcriptomes of cancer and immune cells from metastatic RCC patients that had either been exposure to immune checkpoint blockade (ICB) treatment or not. Following preprocessing, as described in the original publication, the dataset included 34 326 cells and 32 718 detected genes obtained from eight patients. We performed traditional-SAM and block-SAM analyses to compare the gene expression profiles of cancer cells, CD8+ T-cells, and a subset of genes associated with immune evasion and checkpoints between treatment-naïve patients (*n* = 3) and those exposed to ICB therapy (*n* = 5). Analyses were conducted using the normalized UMI counts provided through the portal. SAM was performed using 1000 permutations with a predefined FDR threshold of 1%.

Computational benchmarking revealed that the analysis required ∼104.6 min and reached a peak memory usage of ∼9264 MB (∼9.1 GB), with a net memory increase of ∼1063 MB (∼1.0 GB) over the run. While this is computationally heavy, it is well within the capabilities of a typical modern workstation and is comparable to other permutation-based analyses at this scale, with runtime driven largely by the 1000-permutation budget and the large number of features.

**Figure 8:**
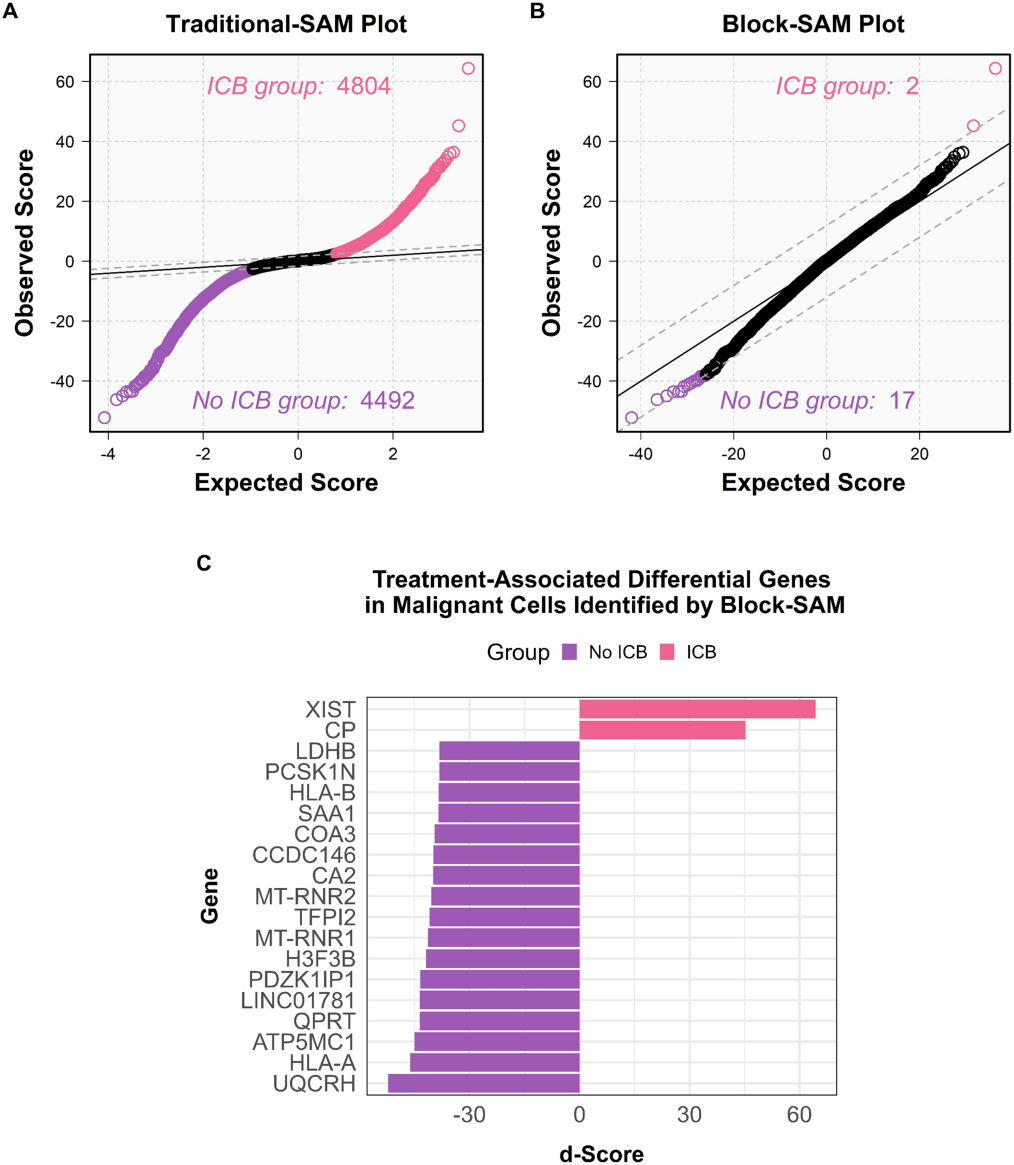
Differentially abundant features in malignant cells associated with ICB treatment exposure status identified by both traditional and block-SAM analyses (*n* = 8 patients; three exposed to treatment, five treatment-naïve). (A) Traditional-SAM plot. Features associated with malignant cells exposed to ICB treatment (*n* = 15 257) and treatment-naïve cancer cells (*n* = 4385) deviate from the null distribution, while non-significant features fall within the threshold bounds (dashed lines). (B) Block-SAM plot. Features associated with cancer cells exposed to ICB treatment (*n* = 2) and treatment-naïve cancer cells (*n* = 17) deviate from the null distribution. (C) Differentially expressed genes in malignant cells identified by block-SAM across ICB treatment exposure status. The identities of genes upregulated in the group exposed to ICB treatment (*n* = 2) and those upregulated in the treatment-naïve group (*n* = 17).

When applying traditional-SAM, 9296 genes were identified as differentially expressed between malignant cells based on exposure to ICB therapy (Fig. 8A). These results imply that ∼28.4% of all genes detected were statistically different between the groups—an implausibly high proportion given that the analysis was based on data from only eight patient samples. Of these, 4804 genes were weighted toward ICB-exposed cells, while 4492 were weighted toward ICB-naïve malignant cells. In contrast, block-SAM identified only 19 genes as differentially expressed between the two treatment exposure groups (Fig. 8B). This represents a substantial reduction in false positives, achieved through the incorporation of the correct sample-level structure by block-SAM.

Note that the large reduction in discoveries when moving from cell-level to patient-level inference does not indicate over-stringency; it reflects correction of pseudoreplication-driven false positives that arise when cells are treated as independent replicates. This distinction is well documented in single-cell DE, where methods that do not account for between-patient variability can yield spurious discoveries even under null conditions (Squair *et al.* 2021, Zimmerman *et al.* 2021). Exploratory discovery can be increased by relaxing the FDR threshold (e.g. 28 genes at 5% FDR and 56 genes at 10% FDR), while retaining valid inference (Fig. S10, available as supplementary data at *Bioinformatics* online).

The identities of the differentially expressed genes are shown in Fig. 8C. Results of the traditional- and block-SAM comparisons of CD8+ T-cells between the treatment exposure groups can be seen in the Supplementary Information (Figs S11 and S12, available as supplementary data at *Bioinformatics* online).

**Figure 9:**
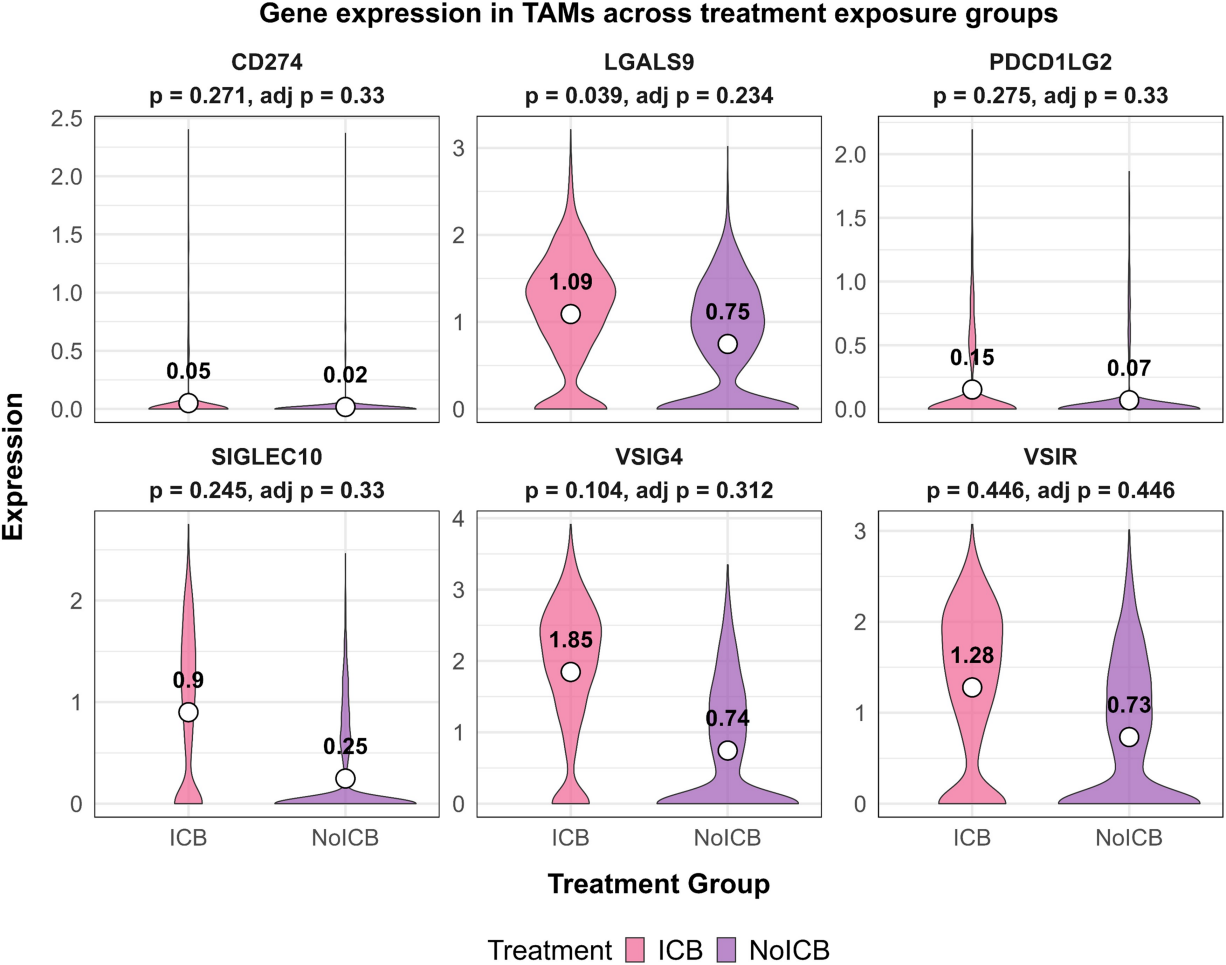
Expression of immune checkpoint and evasion genes within tumor-associated macrophages (TAMs) across the ICB exposure groups. *P*-values were calculated by a block permutation test utilizing the *t*-test statistic and Benjamini–Hochberg correction for multiple comparisons.

A subset of genes involved in immune checkpoint and evasion genes were further investigated by Bi *et al.* (2021). These include CD274, the gene encoding the immune checkpoint protein PD-L1 (Nakayama *et al.* 2024); LGALS9, the gene encoding for galactin-9 known to be involved in regulation of the tumor microenvironment (Lv *et al.* 2023); PDCD1LG2, the gene encoding the immune checkpoint protein PD-L2 (Huang *et al.* 2020), SIGLEC10 which encodes for an immune checkpoint receptor (Yin and Gao 2020); VSIG4, encoding for the protein by the same name responsible for negative regulation of T-cell responses (Li *et al.* 2017); and lastly VSIR which encodes for the VISTA protein, another immune checkpoint protein (Liu *et al.* 2022). All genes were reported to be significantly upregulated in TAMs derived from patients that received ICB therapy compared to those that did not, based on a two-sided Wilcoxon rank-sum test with Bonferroni correction for multiple comparisons. However, this comparison did not account for the nested structure inherent to scRNA-seq data. When expression levels of these genes were reevaluated using a targeted block permutation test utilizing a *t*-test statistic and Benjamini–Hochberg correction (Fig. 9), none of the genes were found to be significantly different between the groups. This underscores the importance of accounting for the block structure of scRNA-seq data, as it greatly reduces false positives when applied in a targeted analysis.

## 4 Conclusion

In this study, we developed and implemented the block-SAM method to analyze high-dimensional data with nested sample structure. Across both MSI and scRNA-seq datasets, block-SAM consistently identified a substantially lower number of statistically significant features with lower false positive rate, when compared to traditional-SAM. Importantly, block-SAM retained all biologically relevant features while significantly reducing spurious findings, demonstrating its robustness across diverse tissue types and experimental conditions, and allowing careful analysis and identification of biologically important molecular features that truly discriminate between groups. Note that block-SAM is currently formulated for designs with a single primary level of biological clustering and does not explicitly accommodate multi-level hierarchies such as technical replicates, longitudinal sampling, or nested batches. Batch effects can be addressed using standard preprocessing prior to analysis, and mixed-effects models remain appropriate when distributional assumptions are tenable, and sample sizes are sufficient. Extending block-SAM to hierarchical permutation schemes that respect multiple levels of nesting is a natural future direction but will require careful definition of the null hypothesis alongside computational and interpretational considerations.

Taken together, the findings described in this article show that block-SAM provides a statistically rigorous approach to analyzing data with a nested structure, while preserving biologically meaningful features. By reducing false discoveries, it streamlines annotation efforts and enhances the reliability of metabolomic insights across multiple cancer types. Given its consistent performance across independent datasets, block-SAM represents a powerful advancement for the statistical analysis of MSI and scRNA-seq datasets.

## Supplementary Material

btag137_Supplementary_Data
