# Supplementary material for: Structure-preserving multivariate hypothesis testing for mass spectrometry imaging and single-cell data
Source: Bioinformatics. 2026 Mar 21;42(4):btag137. doi: 10.1093/bioinformatics/btag137 (PMC13070395; doi:10.1093/bioinformatics/btag137)
Supplement: btag137_Supplementary_Data [file btag137_supplementary_data.docx]

**Supplementary Information**

**Structure-Preserving Multivariate Hypothesis Testing for Mass Spectrometry Imaging and Single-Cell Data**

Keziah E. Liebenberg^1^, Erin Craig*^2,3^, Robert Tibshirani^2^, Livia S. Eberlin^1^*

^1^ Department of Surgery, Baylor College of Medicine, Houston TX

^2^ Department of Biomedical Data Science and Department of Statistics, Stanford University, Stanford CA

^3^ Department of Biostatistics, University of Michigan, Ann Arbor, MI

**to whom correspondence should be addressed:* eberlin@bcm.edu and ercr@umich.edu

**Comparing Block-SAM and Per-Patient SAM Under Equal Pixel Counts**

Block-SAM and per-patient SAM tackle the same dependence problem in different ways. Per-patient SAM first aggregates pixel intensities to patient-level means and then performs inference at the patient level. Block-SAM retains all pixels in the analysis but restricts permutations to the patient labels, thereby preserving within-patient correlation while testing for between-patient differences. When all patients have the same number of pixels and block-SAM uses patient-level variance estimation, these approaches are mathematically equivalent.

A simulation was performed to illustrate this equivalence. Using the kidney cancer dataset, signal was injected into 50 randomly selected features. To enforce equal pixel counts across patients, each image was randomly subset to 246 pixels (the minimum number of pixels observed across images). Block-SAM and per-patient SAM were then compared across a range of signal strengths using 100 simulation replicated per signal level. As expected, we find that per-patient and block-SAM results are nearly identical, with small difference reflecting variability in permutation testing (**Figure S1**).


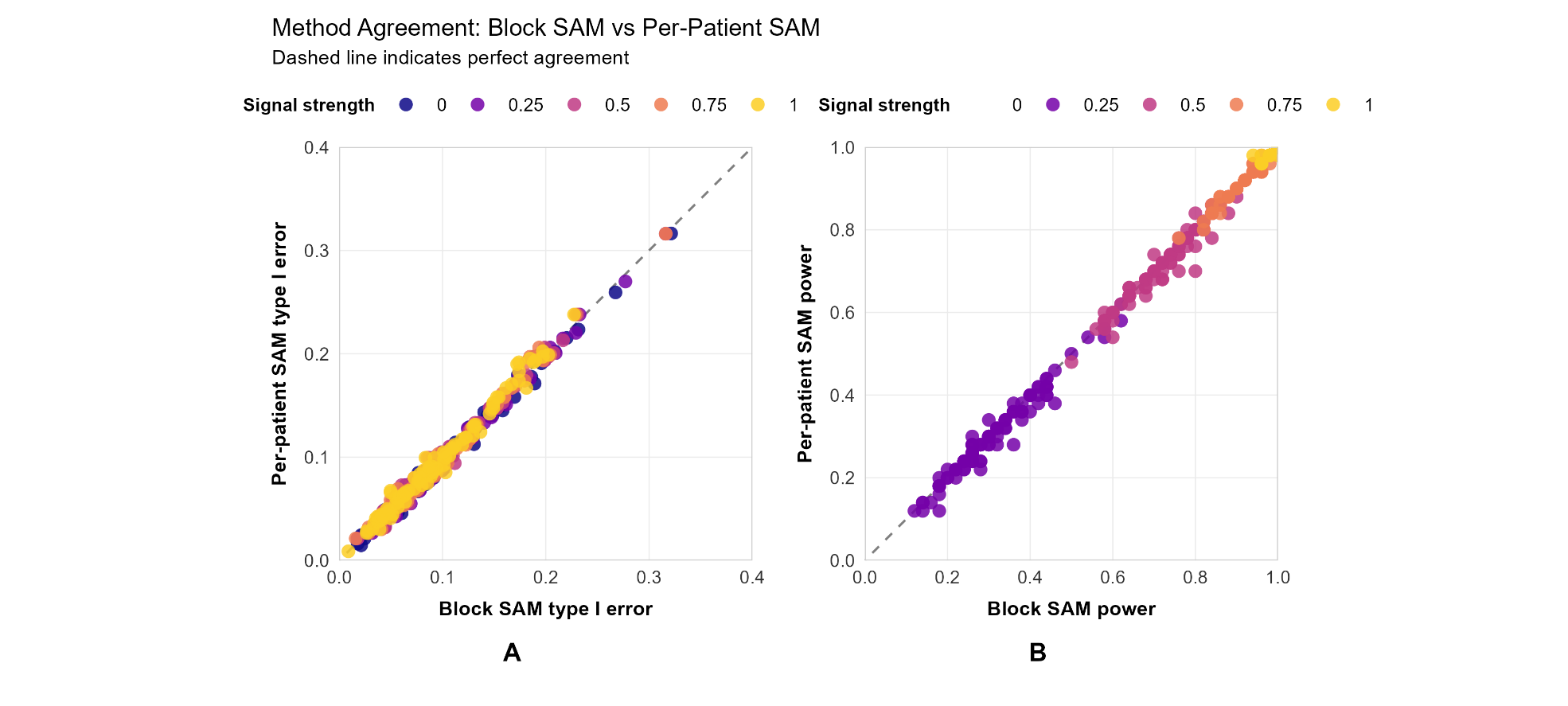


**Figure S1. Agreement Plots Comparing Block-SAM and Per-Patient SAM Under Equal Pixel Counts.** **(A)** Scatterplot of block-SAM vs per-patient SAM **Type I error rates** across signal strengths (dashed line = perfect agreement). **(B)** Scatterplot of block-SAM vs per-patient SAM **statistical power** across signal strengths (dashed line = perfect agreement).

**Evaluation of the Algorithmic Reproducibility of Block-SAM**

To assess the algorithmic reproducibility of block-SAM, the method was run 10 times on the kidney DESI-MSI dataset using different random seeds while keeping all other parameters constant (1,000 permutations, FDR = 1%). For each seed, the set of features selected as significant was recorded. Reproducibility was quantified using pairwise Jaccard similarity coefficients, calculated as the number of features selected by both runs divided by the number selected by either run. A "core set" was defined as features selected in all 10 runs, representing perfectly reproducible differential features. To assess consistency in selection counts, the mean and standard deviation of features selected across seeds was calculated.


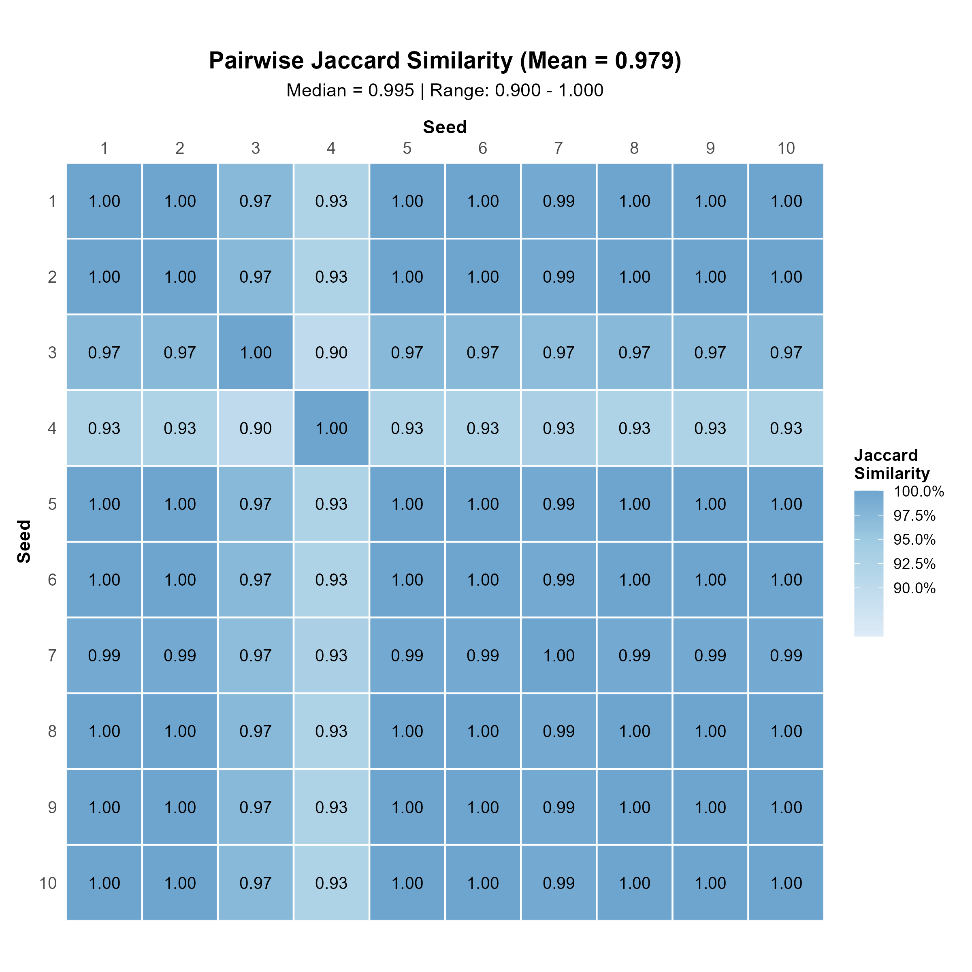


**Figure S2. Reproducibility of block-SAM selected features across seeds.** Across many different random seeds, block-SAM typically selected the same features, with an average pairwise Jaccard similarity of 0.979.

Block-SAM demonstrated exceptional algorithmic reproducibility across random seeds. Pairwise Jaccard similarity between all 10 seed combinations showed a mean of 0.979 (median = 0.995, range: 0.90-1.00), indicating near-identical feature selection regardless of random number generation. The distribution of selection frequencies revealed a clear bimodal pattern: 181 of 201 features (90%) formed a core set selected in all 10 runs, while 15 features (7%) showed sporadic selection in ≤2 runs. Notably, no features fell into an intermediate consistency range (3-8 seeds), indicating a sharp distinction between robust differential signals and methodological noise. Each run selected a comparable number of features (187 ± 5), demonstrating stable algorithm behavior. These results confirm that block-SAM provides highly reproducible feature selection independent of stochastic variation in the permutation testing procedure.

**Evaluation of the Effect of Patient Size on Block-SAM Power**

Consider the extreme case of n=2 patients per group, the maximum number of unique permutations is C(4,2)=6, yielding a minimum achievable p-value of ~0.167. As a result, significance testing at conventional thresholds (α=0.05) is impossible regardless of the statistical method employed—whether permutation-based, parametric (t-test), or non-parametric (Wilcoxon). To study the impact of sample size on statistical power for block-SAM, a systematic evaluation of block-SAM’s performance across a range of sample sizes was conducted using the kidney DESI-MSI dataset.

For each combination of patient count K ∈ {4, 6, 12, 20, 28, 39} and target effect size d ∈ {0.25, 0.5, 0.75, 1}, 100 independent replicates were generated, and the mean power for each (K, d) pair reported. In each replicate, K patients were randomly sampled without replacement from the full cohort (with no patient duplicated within a replicate, though patients could reappear across different replicates). The dashed reference line indicates 80% power. Figure S3 demonstrates an improved block-SAM power with larger cohorts and inherent limitations at very small $n$due to the finite number of unique permutations.


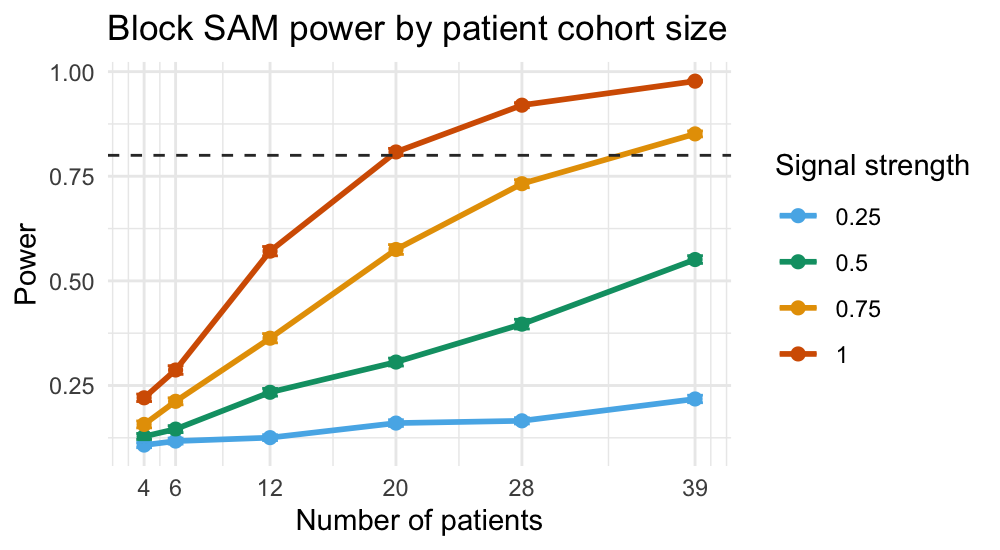


**Figure S3. Impact of patient count and effect size on block-SAM performance.** Signal strength interacts with sample size to improve power.

**Application of Block-SAM to Published Datasets**


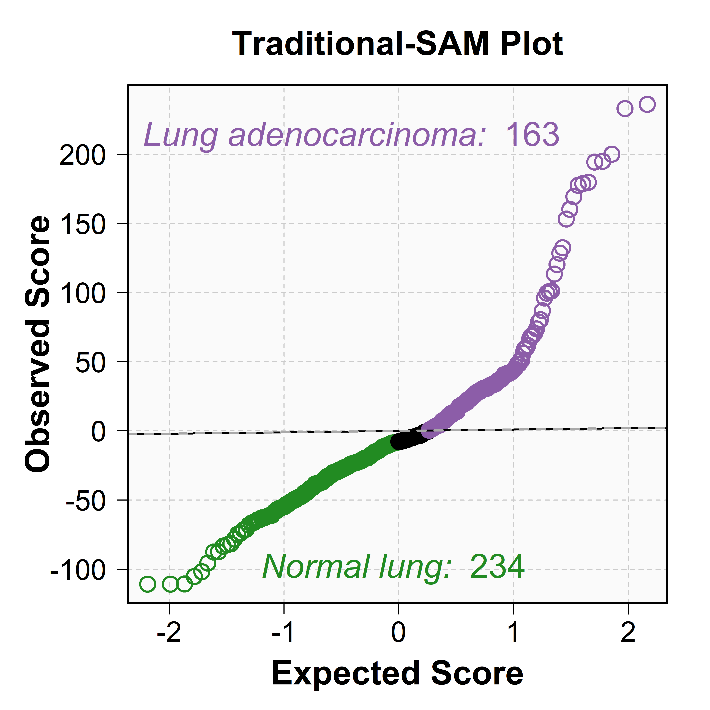

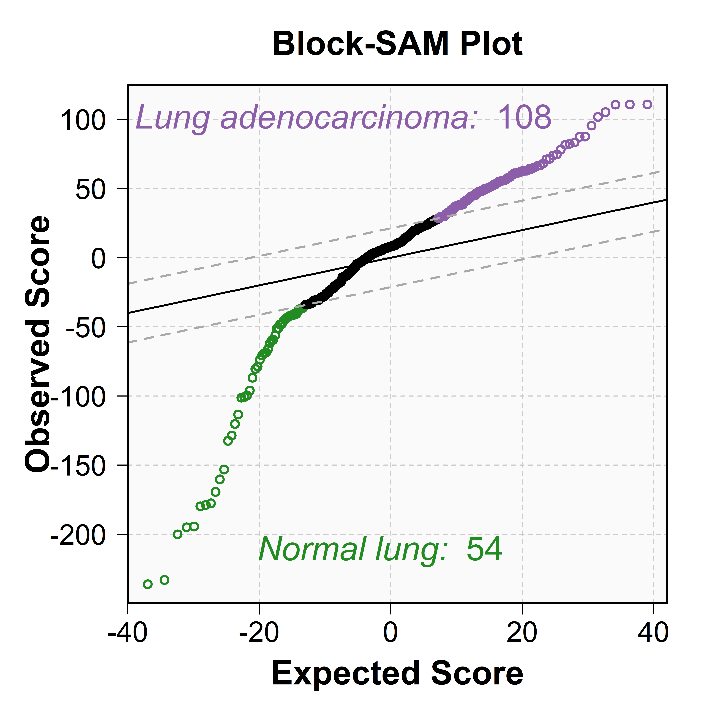


**A**

**B**

**Figure S4. Comparing the number of differentially abundant features detected by traditional and block-SAM applied to the lung adenocarcinoma DESI-MSI dataset.** **(A) Traditional-SAM plot.** Features associated with lung adenocarcinoma (ADC) (purple, n = 163) and normal lung tissue (green, n = 234) deviate from the null distribution, while non-significant features (black) fall within the threshold bounds (dashed lines).

**(B) Block-SAM plot.** Features associated with lung ADC (purple, n = 108) and normal lung tissue (green, n = 54) deviate from the null distribution.

**Table S1.** Comparison of the number of statistically significant features identified by traditional-SAM and block-SAM.

| **Feature Category** | **Number of Features** |
| --- | --- |
| Total Features (Traditional-SAM) | 397 |
| Total Features (Block-SAM) | 162 |
| Common | 162 |
| Unique to Traditional-SAM | 235 |
| Unique to Block-SAM | 0 |


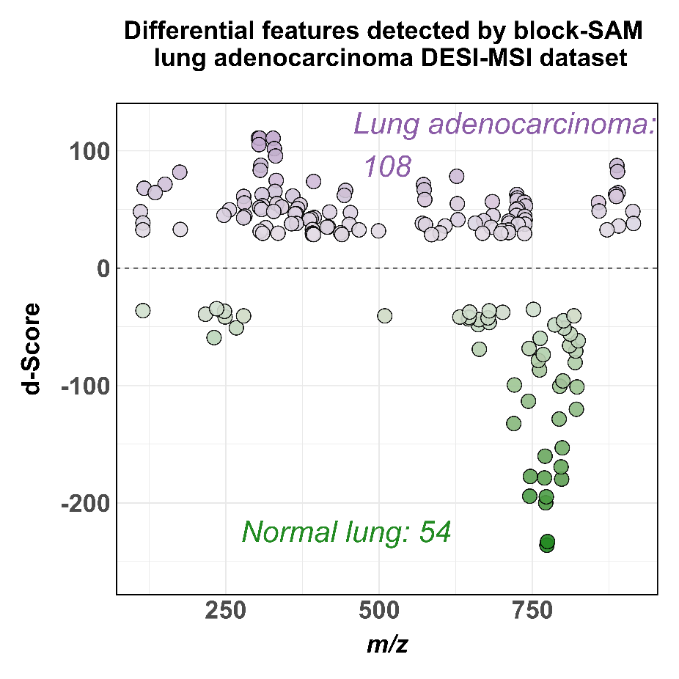

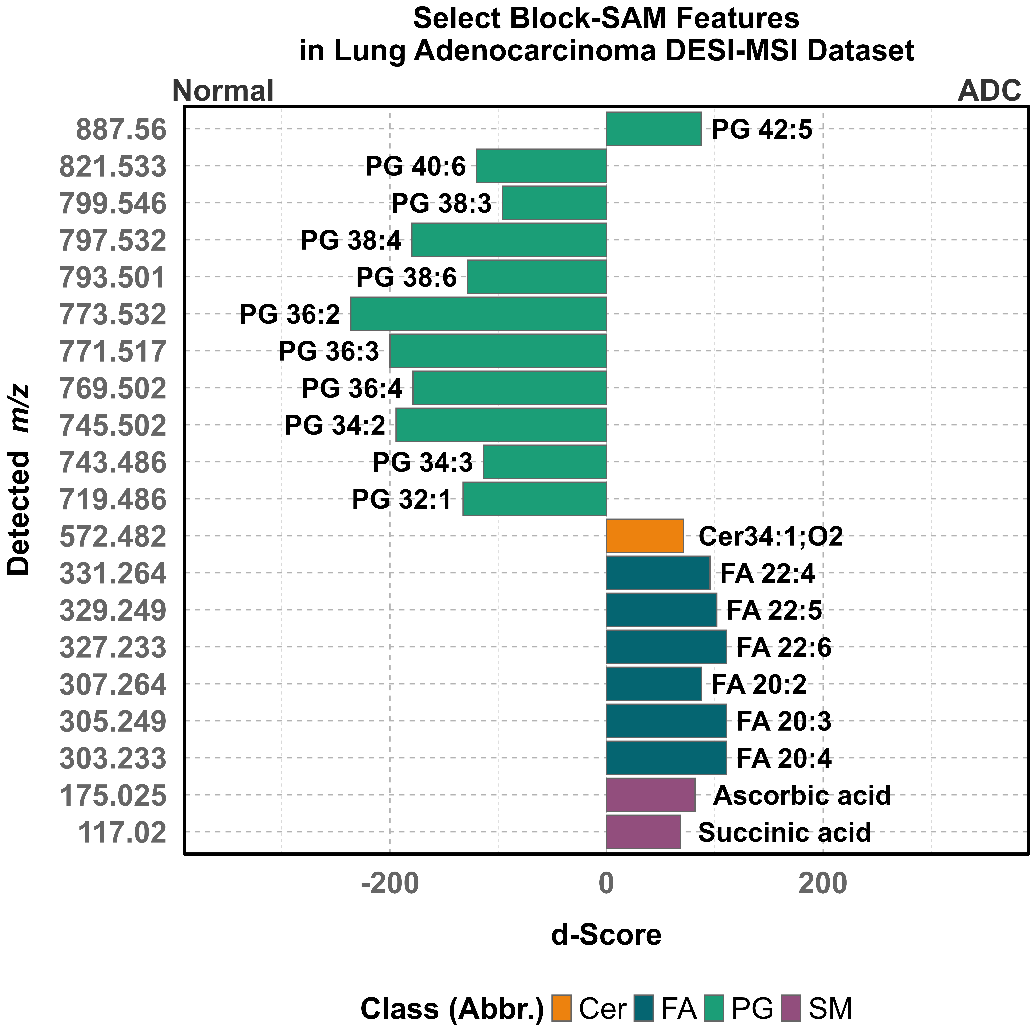


**A**

**B**

**Figure S5. Features selected as differential between normal lung and ADC by block-SAM analysis. (A) Distribution of *m/z* features selected by block SAM as differential.** Features with positive d- Scores (above the dashed line) have a higher significant abundance in ADC tissues, while those with negative d-scores (below the dashed line) are significantly abundant in normal tissues (green). **(B) Tentative annotations of selected differential *m/z* features selected by block-SAM analysis**. Selected features from the block-SAM analysis with tentative molecular annotations, grouped by molecular class. Tentative annotations are displayed accordingly: Molecular class abbreviation, # of carbons: # of double bonds.


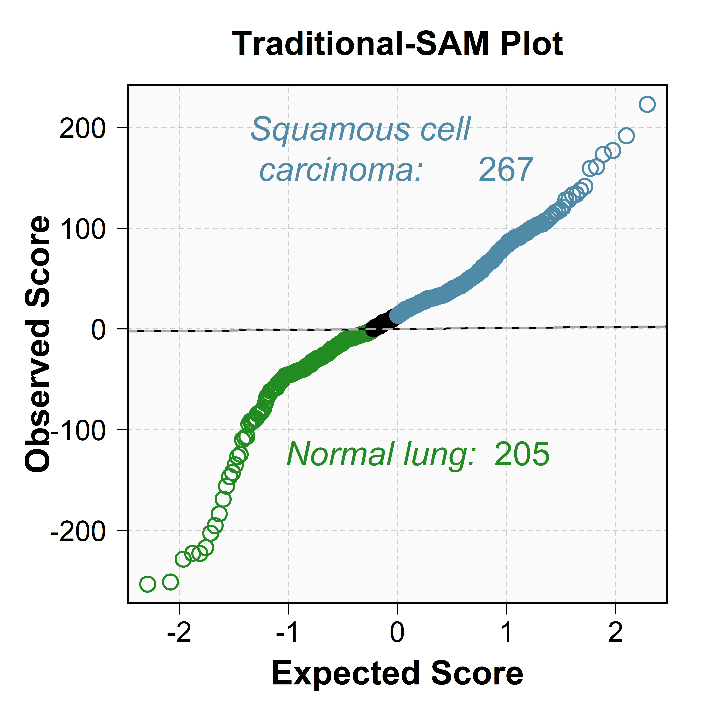

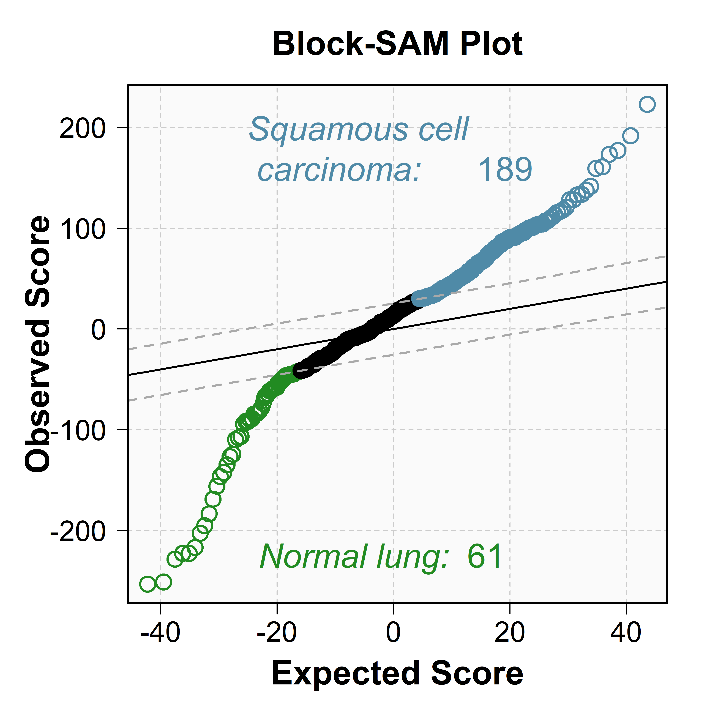


**A**

**B**

**Figure S6. Comparing the number of differentially abundant features detected by traditional and block-SAM applied to the lung squamous cell carcinoma DESI-MSI dataset.** **(A) Traditional-SAM plot.** Features associated with lung squamous cell carcinoma (SCC) (blue, n = 267) and normal lung tissue (green, n = 205) deviate from the null distribution, while non-significant features (black) fall within the threshold bounds (dashed lines). **(B) Block-SAM plot.** Features associated with lung SCC (blue, n = 189) and normal lung tissue (green, n = 61) deviate from the null distribution.

**Table S2.** Comparison of the number of statistically significant features identified by traditional-SAM and block-SAM.

| **Feature Category** | **Number of Features** |
| --- | --- |
| Total Features (Traditional-SAM) | 472 |
| Total Features (Block-SAM) | 250 |
| Common | 250 |
| Unique to Traditional-SAM | 222 |
| Unique to Block-SAM | 0 |


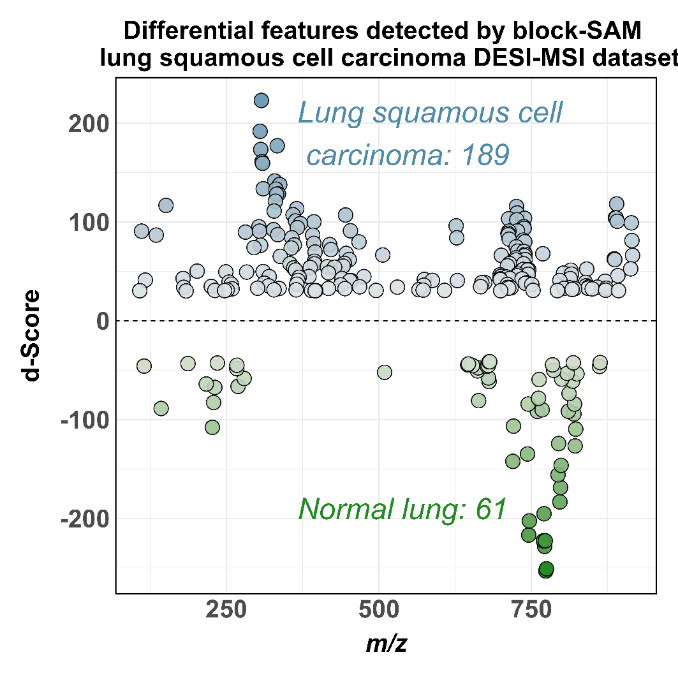

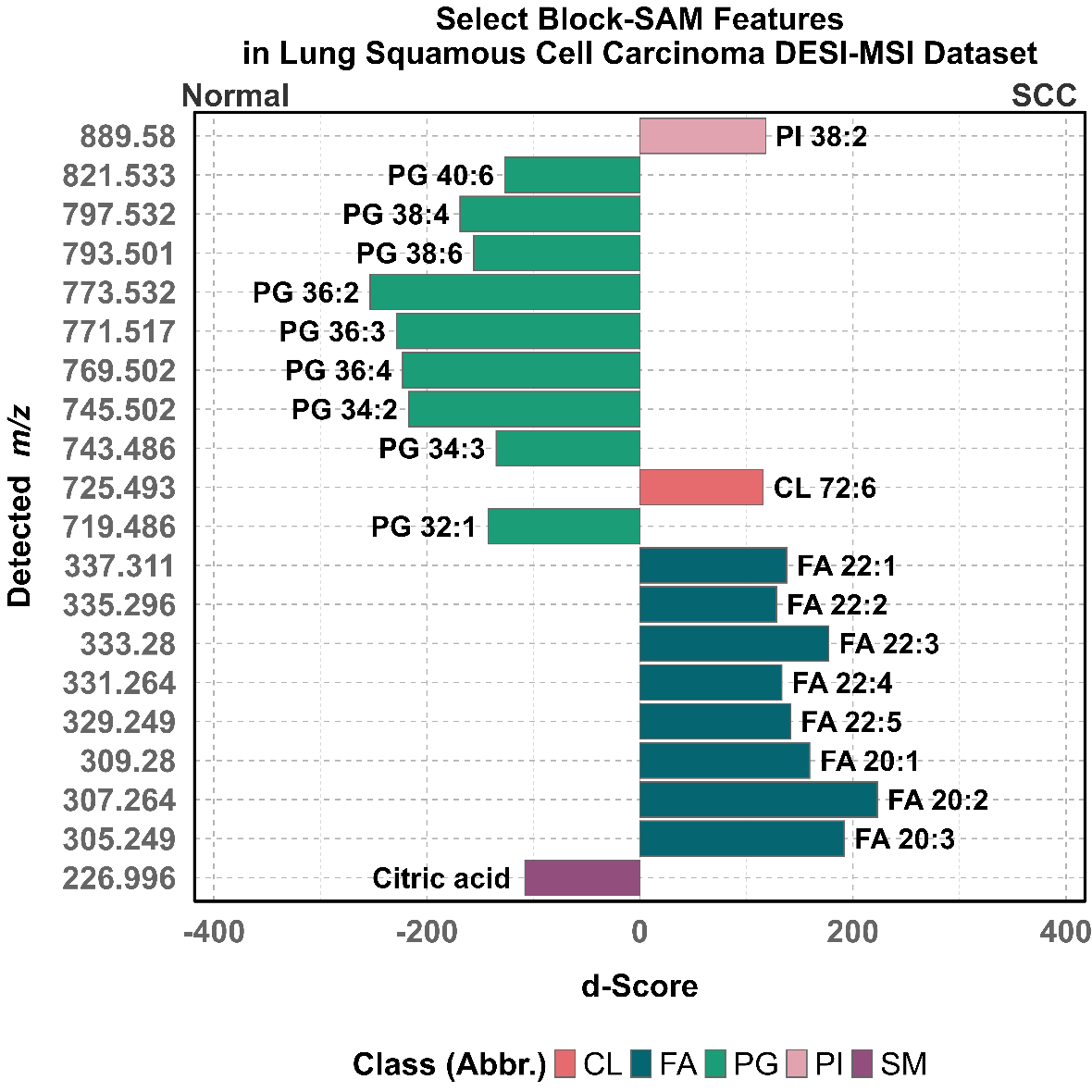


**B**

**A**

**Figure S7. Features selected as differential between normal lung and SCC by block-SAM analysis.**

**(A) Distribution of *m/z* features selected by block SAM as differential in the lung SCC DESI-MSI dataset.** Features with positive d- Scores (above the dashed line) have a higher significant abundance in SCC tissues, while those with negative d-scores (below the dashed line) are significantly abundant in normal tissues (green). **(B) Tentative annotations of selected differential *m/z* features selected by block-SAM analysis**. Selected features from the block-SAM analysis with tentative molecular annotations, grouped by molecular class. Tentative annotations are displayed accordingly: Molecular class abbreviation, # of carbons: # of double bonds.


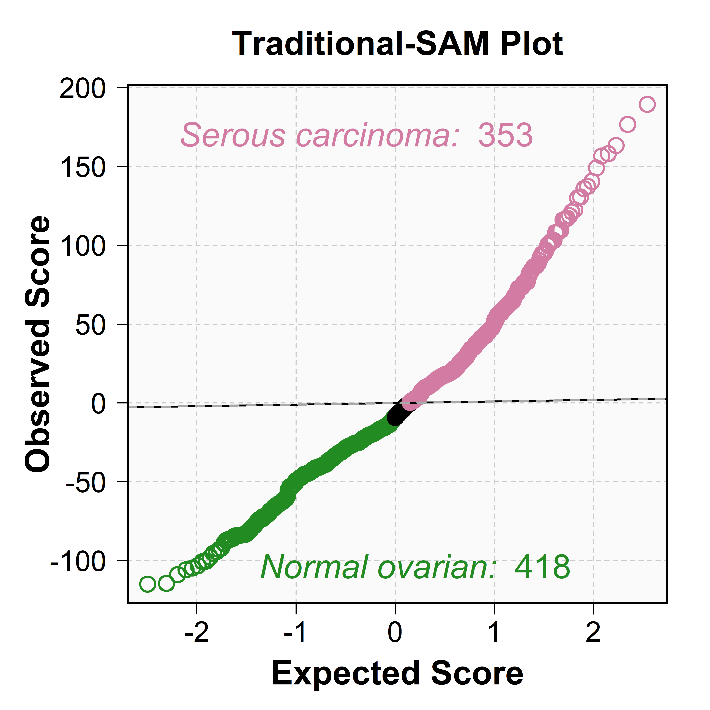

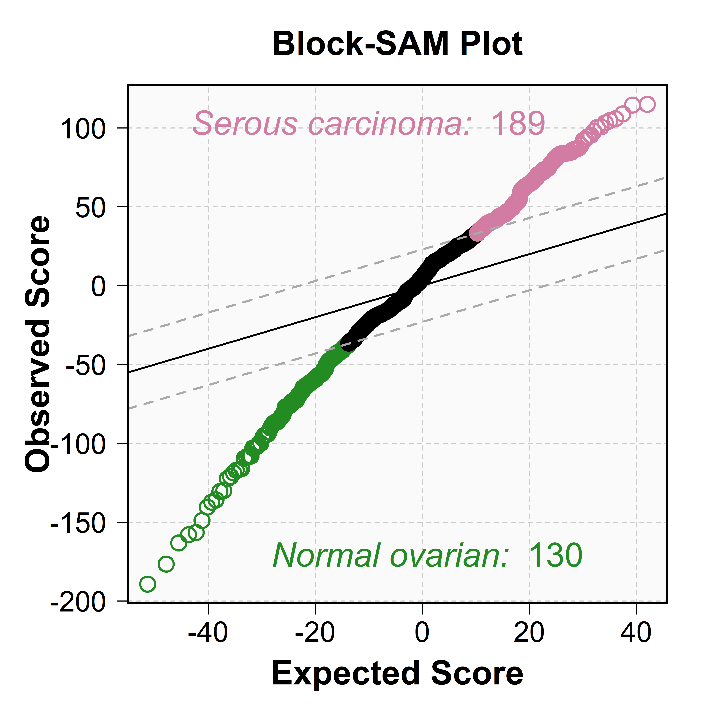


**A**

**B**

**Figure S8. Comparing the number of differentially abundant features detected by traditional and block-SAM applied to the ovarian serous carcinoma DESI-MSI dataset.** **(A) Traditional-SAM plot.** Features associated with ovarian serous carcinoma (pink, n = 353) and normal ovarian tissue (green, n = 418) deviate from the null distribution, while non-significant features (black) fall within the threshold bounds (dashed lines). **(B) Block-SAM plot.** Features associated with ovarian serous carcinoma (pink, n = 189) and normal ovarian tissue (green, n = 130) deviate from the null distribution.

**Table S3.** Comparison of the number of statistically significant features identified by traditional-SAM and block-SAM.

| **Feature Category** | **Number of Features** |
| --- | --- |
| Total Features (Traditional-SAM) | 771 |
| Total Features (Block-SAM) | 319 |
| Common | 319 |
| Unique to Traditional-SAM | 452 |
| Unique to Block-SAM | 0 |


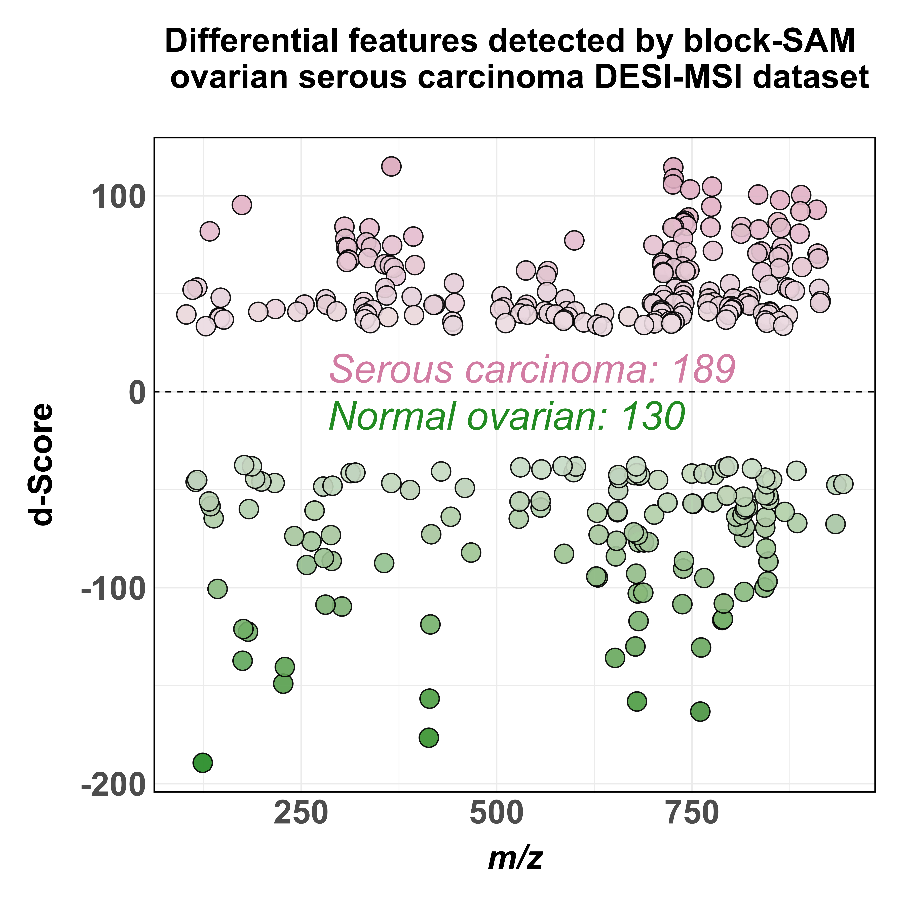


**A**


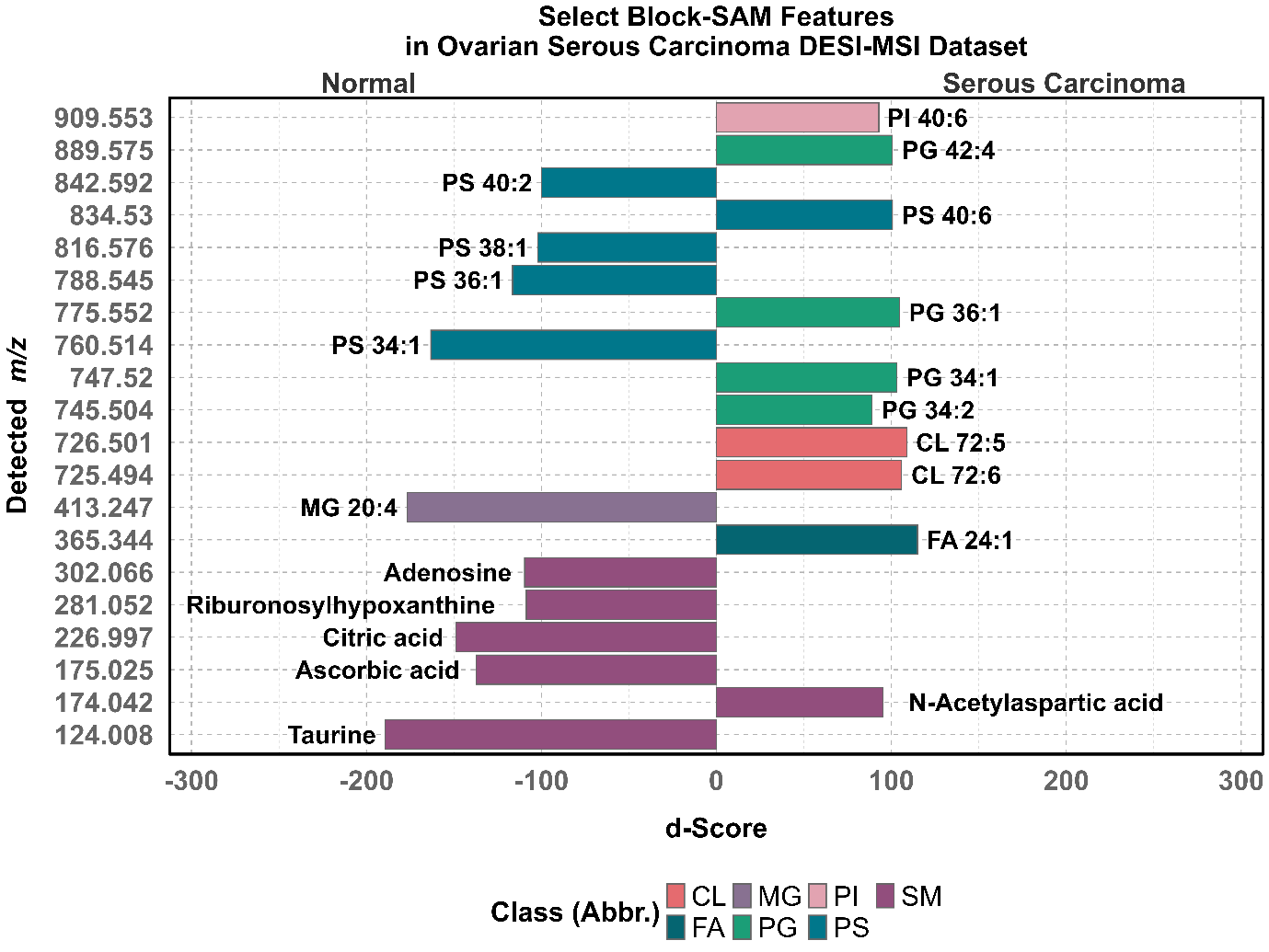


**B**

**Figure S9. Features selected as differential between normal ovarian and serous carcinoma by block-SAM analysis. (A) Distribution of *m/z* features selected by block SAM as differential in the ovarian serous carcinoma DESI-MSI dataset.** Features with positive d- Scores (above the dashed line) have a higher significant abundance in serous carcinoma tissues, while those with negative d-scores (below the dashed line) are significantly abundant in normal tissues (green). **(B) Tentative annotations of selected differential *m/z* features selected by block-SAM analysis**. Selected features from the block-SAM analysis with tentative molecular annotations, grouped by molecular class. Tentative annotations are displayed accordingly: Molecular class abbreviation, # of carbons: # of double bonds.

**Adjusting Statistical Stringency: FDR Thresholds vs. Pseudoreplication**

It is important to note that the perceived reduction in features selected by block-SAM in comparison to traditional-SAM when applied to scRNA-seq data does not indicate that block-SAM is overly conservative or missing important signals. Rather, it reflects correction of massive false positive inflation that occurs when cells are incorrectly treated as independent observations. For researchers concerned about statistical power, the solution is adequate patient replication (n≥10 per group), not relaxation of statistical stringency through pseudoreplication. For users concerned about maximizing discovery in exploratory analyses, the FDR threshold can be adjusted as seen in the figure below.


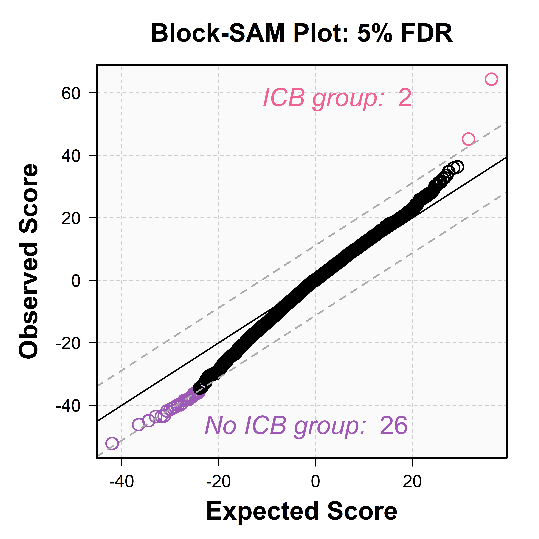

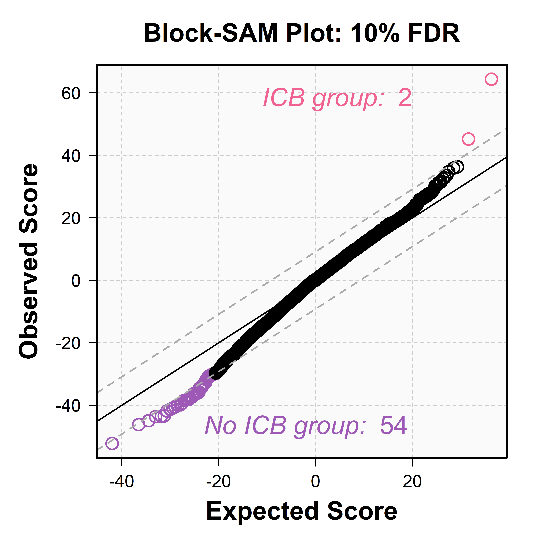

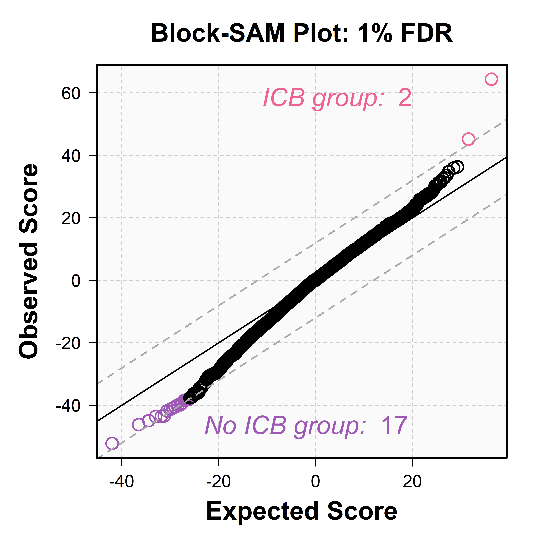


**A**

**B**

**C**

**Figure S10. Comparison of the number of features selected as significant by block-SAM under varying FDR selections. (A)** Block-SAM plot under **1% FDR contro**l, 19 features selected. **(B)** Block-SAM plot under 5% FDR control, 28 features selected. **(C)** Block-SAM plot under 10% FDR control, 56 features selected.


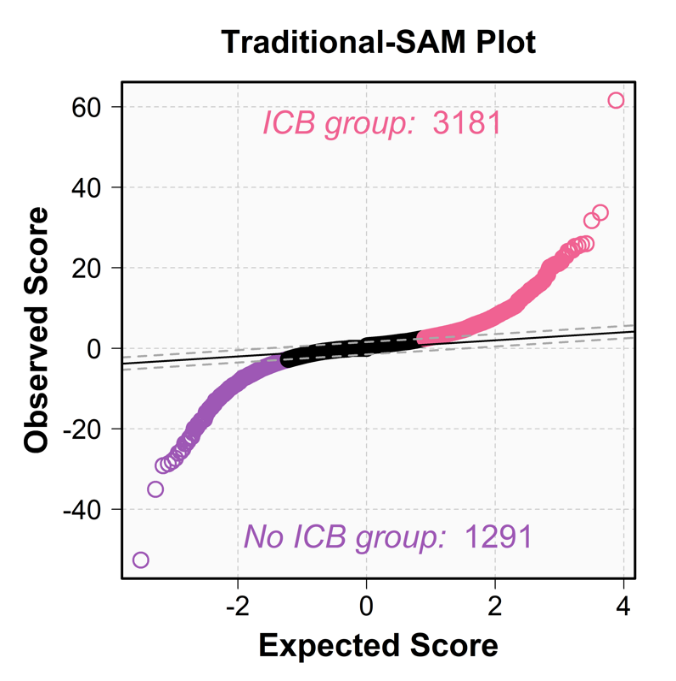

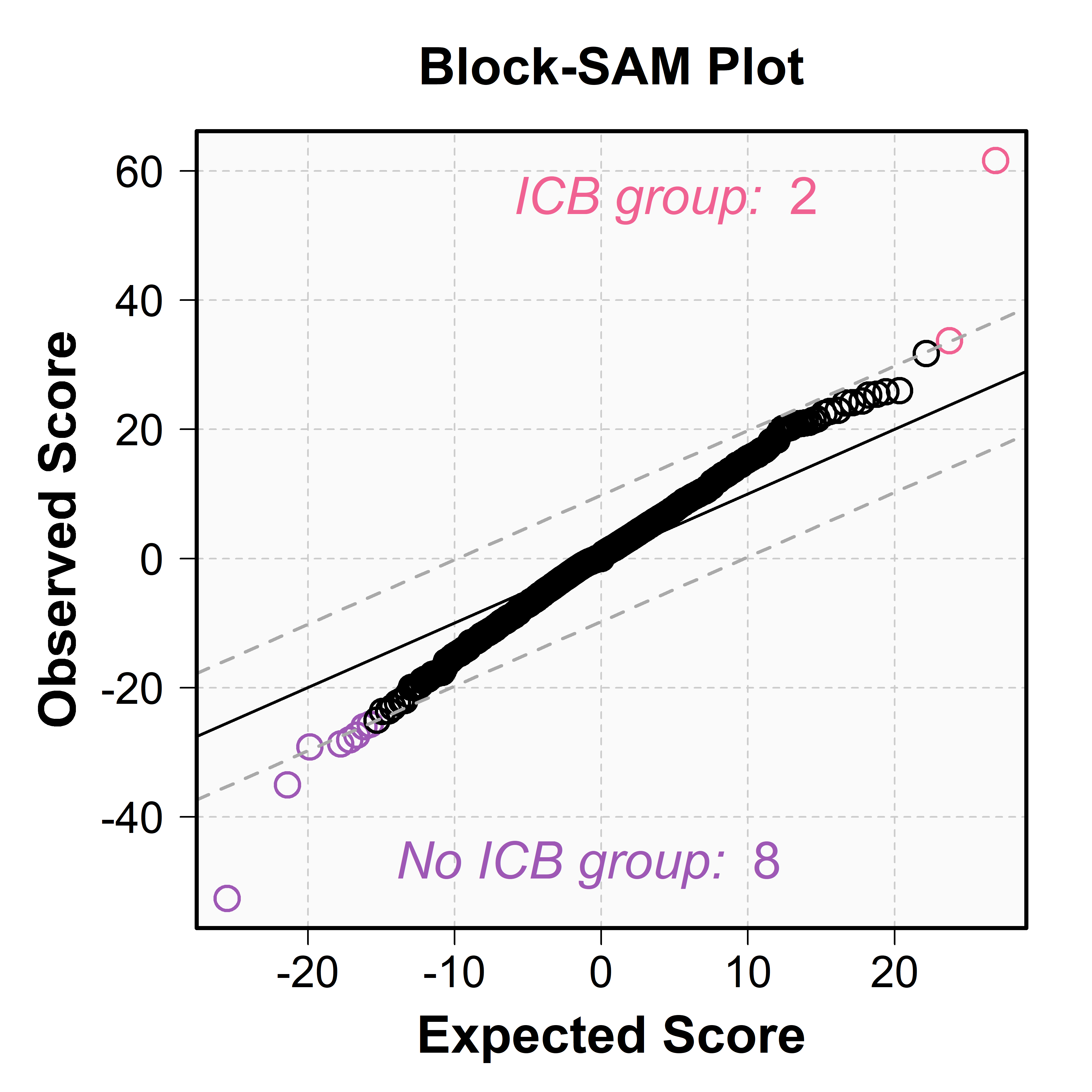


**B**

**A**

**Figure S11. Differentially abundant features in CD8+ T-cells associated with ICB treatment exposure status identified by both traditional and block-SAM analyses (n = 8 patients; 3 exposed to treatment, 5 treatment-naïve).** **(A) Traditional-SAM plot.** Features associated with malignant cells exposed to ICB treatment (pink, n = 3,181) and treatment-naïve cancer cells (purple, n = 1,291) deviate from the null distribution, while non-significant features (black) fall within the threshold bounds (dashed lines). **(B) Block-SAM plot.** Features associated with cancer cells exposed to ICB treatment (pink, n = 2) and treatment-naïve cancer cells (purple, n = 8) deviate from the null distribution.


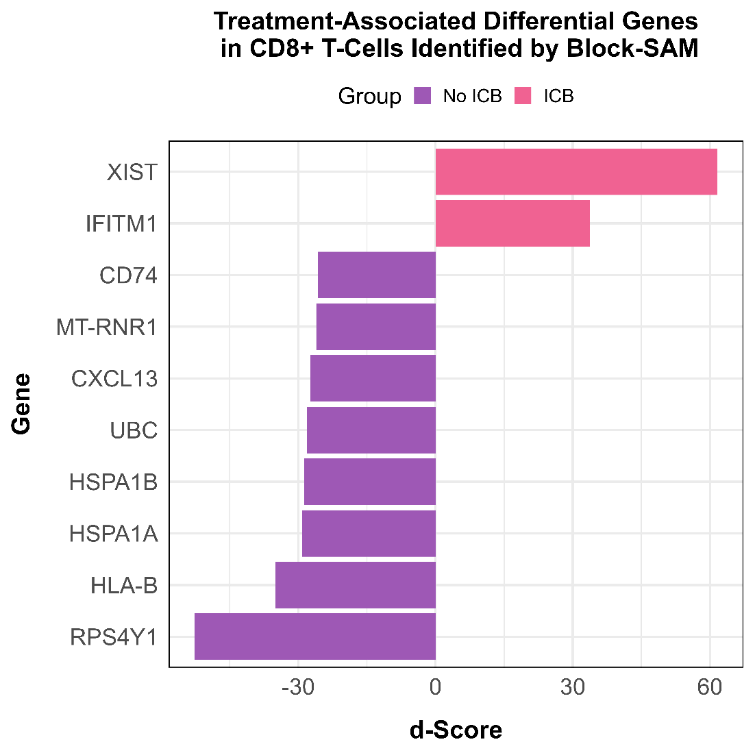


**Figure S12. Differentially expressed genes in CD8+ T-cells identified by block-SAM across ICB treatment exposure status.** The identities of genes upregulated in the group exposed to ICB treatment (pink, n = 2) and those upregulated in the treatment-naïve group (purple, n = 8).
